# Induction of Split Anergy Conditions Natural Killer Cells to Promote Differentiation of Stem Cells through Cell–Cell Contact and Secreted Factors

**DOI:** 10.3389/fimmu.2014.00269

**Published:** 2014-06-19

**Authors:** Han-Ching Tseng, Vickie Bui, Yan-Gao Man, Nicholas Cacalano, Anahid Jewett

**Affiliations:** ^1^Division of Oral Biology and Oral Medicine, The Jane and Jerry Weintraub Center for Reconstructive Biotechnology, Los Angeles, CA, USA; ^2^Bon Secours Cancer Institute, Bon Secours Health System, Richmond, VA, USA; ^3^Department of Radiation Oncology, University of California Los Angeles School of Medicine, Los Angeles, CA, USA; ^4^The Jonsson Comprehensive Cancer Center, University of California Los Angeles School of Dentistry and Medicine, Los Angeles, CA, USA

**Keywords:** IFN-γ, NK, OSCSCs, OSCCs, MP2, cytotoxicity, regulatory NK

## Abstract

In this paper, we provide evidence that anergized NK cells through secreted factors and direct cell–cell contact have the ability to induce differentiation of healthy dental pulp stem cells and stem cell of apical papillae as well as transformed oral squamous cancer stem cell (OSCSC) and Mia-Paca-2, poorly differentiated stem-like pancreatic tumors, resulting in their resistance to NK cell-mediated cytotoxicity. Induction of NK cell resistance and differentiation in the stem cells correlated with the increased expression of CD54, B7H1, and MHC class I, and mediated by the combination of membrane-bound or secreted IFN-γ and TNF-α from the NK cells since antibodies to both cytokines and not each one alone were able to inhibit differentiation or resistance to NK cells. Similarly, antibodies to both TNF-α and IFN-γ were required to prevent NK-mediated inhibition of cell growth, and restored the numbers of the stem cells to the levels obtained when stem cells were cultured in the absence of anergized NK cells. Interestingly, the effect of anti-IFN-γ antibody in the absence of anti-TNF-α antibody was more dominant for the prevention of increase in surface receptor expression since its addition abrogated the increase in CD54, B7H1, and MHC class I surface expression. Antibodies to CD54 or LFA-1 was unable to inhibit differentiation whereas antibodies to MHC class I but not B7H1 increased cytotoxicity of well-differentiated oral squamous carcinoma cells as well as OSCSCs differentiated by the IL-2 + anti-CD16 mAb-treated NK cells whereas it inhibited the cytotoxicity of NK cells against OSCSCs. Thus, NK cells may inhibit the progression of cancer by killing and/or differentiation of cancer stem cells, which severely halt cancer growth, invasion, and metastasis.

## Introduction

Recent advances in our understanding of anti-tumor immune responses and cancer biology have revealed a complex dynamic interaction between the immune effectors and the tumor cells. Effectors of the immune system are known to shape tumor cells (immuno-editing) and select for cancers with reduced immunogenicity and enhanced capacity to actively induce immunosuppression. However, the same effector mechanisms are likely responsible for the selection of healthy stem cells with enhanced capacity to induce immunosuppression for the ultimate goal of wound healing, tissue regeneration, and cessation of inflammation. Much work has been done to identify strategies by which tumor cells evade the immune system. Altered expression of MHC class I molecules which modulate the function of T and NK cells are one of the examples of such mechanism. In addition, tumor cells induce T and NK cell apoptosis, block lymphocyte homing and activation, and dampen macrophage and dendritic cell function by releasing immunosuppressive factors such as Fas, VEGF, IL-6, IL-10, TNF-α, GM-CSF, and IL-1β. Many factors responsible for the suppression of NK cell cytotoxicity in humans have been previously identified (1–6). It has been shown that freshly isolated tumor infiltrating NK cells are not cytotoxic to autologous tumors. Moreover, NK and T cells obtained from the peripheral blood of patients with cancer have significantly reduced cytotoxic activity (7–14). In addition, NK cell cytotoxicity is suppressed after their interaction with stem cells (15–17).

We have previously shown that K562, an NK-sensitive tumor, causes loss of NK cell cytotoxicity while increasing IFN-γ secretion by the NK cells, and induces cell death in a small subset of NK cells (18, 19). On the other hand, NK-resistant tumors such as RAJI cells do not induce loss of NK cell cytotoxicity nor IFN-γ secretion (18, 19). Furthermore, following NK cell cultures with sensitive tumor-target cells overnight, the target binding NK cells undergo phenotypic and functional changes expressing CD16−CD56+/dim/−CD69+ phenotype (18, 19). Significant down-modulation of CD16 receptor expression and decreased NK cell cytotoxic function were also seen in cancer patients including those who suffer from oral and ovarian cancers (20, 21). In addition, down-regulation of CD16 surface receptors on NK cells was also observed when NK cells were treated with CA125 isolated from ovarian tumor cells (22). The decrease in CD16 surface receptors was accompanied by a major decrease in NK cell killing activity against K562 tumor cells (22). These observations suggested that CD16 receptors may play an important role in target cell-induced loss of NK cell cytotoxicity. Furthermore, we have previously shown that triggering of CD16 on NK cells was found to result in down-modulation of CD16 receptors and in a great loss of cytotoxicity and augmented secretion of IFN-γ, which we have previously coined as “split anergy” (18, 19, 23–27). Loss of cytotoxicity in NK cells was significantly increased when NK cells were either treated with anti-MHC class I antibody (25) or treated with F(ab)′_2_ fragment of anti-CD16 mAb (25, 27).

Progress has been made in identification of the upstream mechanisms, which control the expression of immunosuppressive factors in tumor cells. Two key control elements, NFκB and STAT3 were shown to regulate coordinately the production of multiple tumor-derived immunosuppressive molecules and play a pivotal role in tumor cell immune suppression.

The significance and exact mechanisms by which NFκB nuclear function in oral tumors modulate and shape the function of key interacting immune effectors is starting to unravel. We have previously shown that differentiated primary oral epithelial tumors, unlike their cancer initiating cells, demonstrate higher nuclear NFκB activity and secrete significant levels of cytokines and chemokines, and are resistant to NK cell-mediated cytotoxicity (28, 29). Moreover, inhibition of NFκB in differentiated oral squamous carcinoma cells (OSCCs), or in non-tumorigenic oral keratinocytes (HOK-16B) leads to a significant increase in NK cell-mediated cytotoxicity and secretion of IFN-γ (30, 31). In addition, targeted inhibition of NFκB in skin epithelial cells resulted in the induction of auto-immunity and inflammation (32). Also, blocking of NFκB function in both the intestinal epithelial cells and myeloid cells was previously shown to result in a significant decrease in size and numbers of the tumor cells (33).

Our previous studies indicated that the stage of maturation and differentiation of healthy untransformed stem cells, as well as transformed tumorigenic cancer stem cells, is predictive of their sensitivity to NK cell lysis. In this regard, we have shown that oral squamous cancer stem cells (OSCSCs), which are stem-like oral tumors, are significantly more susceptible to NK cell-mediated cytotoxicity; whereas, their differentiated counterpart OSCCs is significantly more resistant (29). In addition, hESCs and hiPSCs, as well as a number of other healthy normal stem cells such as hMSCs and hDPSCs, were found to be significantly more susceptible to NK cell-mediated cytotoxicity than their differentiated counterparts (29). Based on these results, we proposed that NK cells may play a significant role in differentiation of the cells by providing critical signals via secreted cytokines as well as direct cell–cell contact. In addition, we have shown previously that CD14+HLADR− monocytes can condition NK cells to lose cytotoxicity and secrete inflammatory cytokines (34–38). The signals received from the stem cells or monocytes alter the phenotype of NK cells and cause NK cells to lose cytotoxicity and change into cytokine-producing cells. These alterations in NK cell effector function are thought to ultimately aid in driving differentiation of a minor population of surviving, healthy, as well as transformed stem cells. In this paper, we demonstrate that anergized NK cells contribute to differentiation and subsequent resistance of stem cells to NK cell-mediated cytotoxicity through cell–cell contact and secreted cytokines.

## Materials and Methods

### Cell lines, reagents, and antibodies

RPMI 1640 supplemented with 10% fetal bovine serum (FBS) (Gemini Bio-Products, CA, USA) was used for the cultures of human NK cells and monocytes. OSCCs and stem-like OSCSCs were isolated from oral cancer patient tongue tumors at UCLA, and cultured in RPMI 1640 supplemented with 10% FBS (Gemini Bio-Products, CA, USA), 1.4% antibiotic antimycotic, 1% sodium pyruvate, 1.4% non-essential amino acids, 1% l-glutamine, 0.2% gentamicin (Gemini Bio-Products, CA, USA), and 0.15% sodium bicarbonate (Fisher Scientific, PA, USA). Mia-Paca-2 (MP2) were cultured in DMEM with 10% FBS and 1% penicillin and streptomycin (Gemini Bio-Products, CA, USA). Dental pulp stem cells (DPSCs) and stem cell of apical papillae (SCAP) were cultured in DMEM complete medium supplemented with 2% FBS and 1% penicillin and streptomycin (Gemini Bio-Products, CA, USA). To induce differentiation of DPSCs, they were cultured with DMEM in the presence of ascorbic acid (50 μg/ml), Na-β-glycerophosphate (10 mM) (Sigma Aldrich, MO, USA), and dexamethasone (10^−8^ M).

Recombinant IL-2 was obtained from NIH-BRB. Recombinant TNF-α and IFN-γ were obtained from BioLegend (San Diego, CA, USA). The anti-B7H1 was a generous gift from Dr. Liping Chen. Antibodies to CD16, CD54, and LFA-1 were purchased from BioLegend (San Diego, CA, USA). Anti-MHC class I were prepared in our laboratory and 1:100 dilution was found to be the optimal concentration to use. PE – anti-CD26, anti-CD54, anti-CD44, anti-B7H1, anti-CD166, anti-CD326, and anti-CD338 were obtained from BioLegend (San Diego, CA, USA). Antibodies to TNF-α and IFN-γ were prepared in our laboratory and 1:100 dilution was found to be the optimal concentration to use. The human NK and monocyte purification kits were obtained from Stem Cell Technologies (Vancouver, BC, Canada). Cisplatin was obtained through Ronald Reagan Pharmacy at UCLA. Monensin was purchased through BioLegend (San Diego, CA, USA). Propidium iodide (PI) is purchased from Sigma Aldrich (Buffalo, NY, USA).

### Purification of NK cells and monocytes

PBMCs from healthy donors were isolated as described before (19). Briefly, peripheral blood lymphocytes were obtained after Ficoll–hypaque centrifugation and purified NK cells were negatively selected by using an NK cell isolation kit (Stem Cell Technologies, Vancouver, BC, Canada). The purity of NK cell population was found to be >90% based on flow cytometric analysis of anti-CD16 antibody-stained cells. The levels of contaminating CD3+ T cells remained low, at 2.4 ± 1%, similar to that obtained by the non-specific staining using isotype control antibody throughout the experimental procedures. The adherent subpopulation of PBMCs was detached from the tissue culture plates and the total population of monocytes was purified using isolation kit obtained from Stem Cell Technologies (Vancouver, BC, Canada). Greater than 95% purity was achieved based on flow cytometric analysis of CD14 and CD16 antibody-stained monocytes. Written informed consents approved by UCLA Institutional Review Board (IRB) were obtained from the blood donors and all the procedures were approved by the UCLA IRB.

### ELISA and multiplex cytokine array kit

Single ELISAs were performed as described previously (19). Fluorokine MAP cytokine multiplex kits were purchased from R&D Systems (Minneapolis, MN, USA) and the procedures were conducted as suggested by the manufacturer. To analyze and obtain the cytokine concentration, a standard curve was generated by either two or threefold dilution of recombinant cytokines provided by the manufacturer. Analysis was performed using the Star Station software.

### Surface staining and cell death assays

Staining was performed by labeling the cells with antibodies or PI as described previously (19, 24, 39).

### ^51^Cr release cytotoxicity assay

The ^51^Cr release assay was performed as described previously (31). Briefly, different numbers of purified NK cells were incubated with ^51^Cr-labeled tumor-target cells. After a 4-h incubation period, the supernatants were harvested from each sample and counted for released radioactivity using the gamma counter. The percentage specific cytotoxicity was calculated as follows:

$$\%\;\text{Cytotoxicity}=\frac{\text{Experimental}\;\text{cpm}-\text{spontaneous}\;\text{cpm}}{\text{Total}\;\text{cpm}-\text{spontaneous}\;\text{cpm}}$$

LU 30/10^6^ is calculated by using the inverse of the number of effector cells needed to lyse 30% of tumor-target cells × 100.

### Stem cell differentiation with NK cell supernatant

Human NK cells were purified from healthy donor’s PBMCs as described above. NK cells were left untreated or treated with anti-CD16 mAb (3 μg/ml), IL-2 (1000 units/ml), or a combination of IL-2 (1000 units/ml) and anti-CD16 mAb (3 μg/ml) for 18–24 h before the supernatants were removed and used in differentiation experiments. The amounts of IFN-γ produced by activated NK cells were assessed with IFN-γ ELISA (BioLegend, CA, USA). Differentiation of OSCSCs was conducted with gradual daily addition of increasing amounts of NK cell supernatant. On average, a total of 1500 pg of IFN-γ containing supernatants obtained from IL-2 + anti-CD16 mAb-treated NK cells was added for 5 days to induce differentiation and resistance of OSCSCs to NK cell-mediated cytotoxicity. DPSCs and SCAP cells required on average a total of 3600 pg of IFN-γ containing supernatants obtained from IL-2 + anti-CD16 mAb-treated NK cells during a 7-day treatment, whereas MP2 tumors required a total of 7000 pg of IFN-γ containing supernatants from IL-2 + anti-CD16 mAb-treated NK cells for 7 days to promote differentiation and resistance to NK cell-mediated cytotoxicity. Afterward, target cells were rinsed with 1× PBS, detached, and used for experiments.

### Stem cell differentiation with 2% paraformaldehyde-fixed NK cells

Human NK cells were purified as described above. NK cells were left untreated or treated with anti-CD16 mAb (3 μg/ml), IL-2 (1000 units/ml), or a combination of IL-2 (1000 units/ml) and anti-CD16 mAb (3 μg/ml) for 18–24 h. Afterward, supernatants were removed and the NK cells were fixed with freshly prepared 2% paraformaldehyde for 15 min. NK cells were then rinsed three times with 1× PBS and added to tumor cultures. Differentiation of OSCSCs was conducted on average in 5 days with daily and gradual addition of increasing amounts of fixed NK cells. During the differentiation process of OSCSCs, NK cells were added to tumor cells at an effector to target ratio of 0.75–1 for 5 days. For the differentiation of DPSCs, an effector to target ratio of 2:1 was added to culture for 7 days. After the treatment period, NK cells were removed from the tumors and the target cells were used for experiments.

### Treatment of NK cells with monensin

NK cells were purified from healthy donor’s PBMCs as described above. NK cells were left untreated or treated with IL-2 (1000 units/ml) and anti-CD16 mAb (3 μg/ml) with or without monensin (1:1300) for 24 h as per manufacturer’s recommendation. Afterward, the supernatants were harvested from NK cells and the amounts of IFN-γ produced were measured with IFN-γ ELISA (BioLegend, CA, USA). NK cells were then fixed with freshly prepared 2% paraformaldehyde for 15 min and then washed three times with 1× PBS. Thereafter, fixed NK cells were added to OSCSCs at an effector to target ratio of 0.75–1. After 5 days of treatment, NK cells were removed from culture and OSCSCs were used for experiments.

### Statistical analysis

An unpaired, two-tailed Student *t*-test was performed for the statistical analysis. One-way ANOVA with a Bonferroni post-test was used to compare the different groups.

## Results

### Resistance of differentiated OSCCs but not stem-like OSCSCs and DPSCs to NK cell-mediated cytotoxicity; loss of NK cell cytotoxicity and gain in secretion of IFN-γ after NK cell receptor signaling

**Figure 1:**
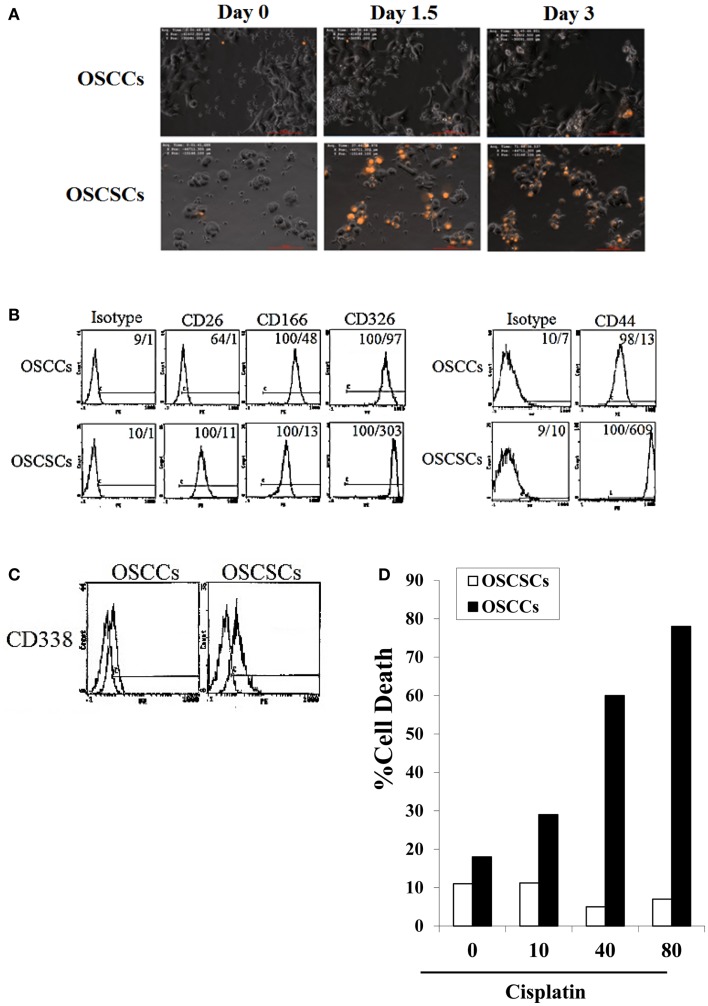

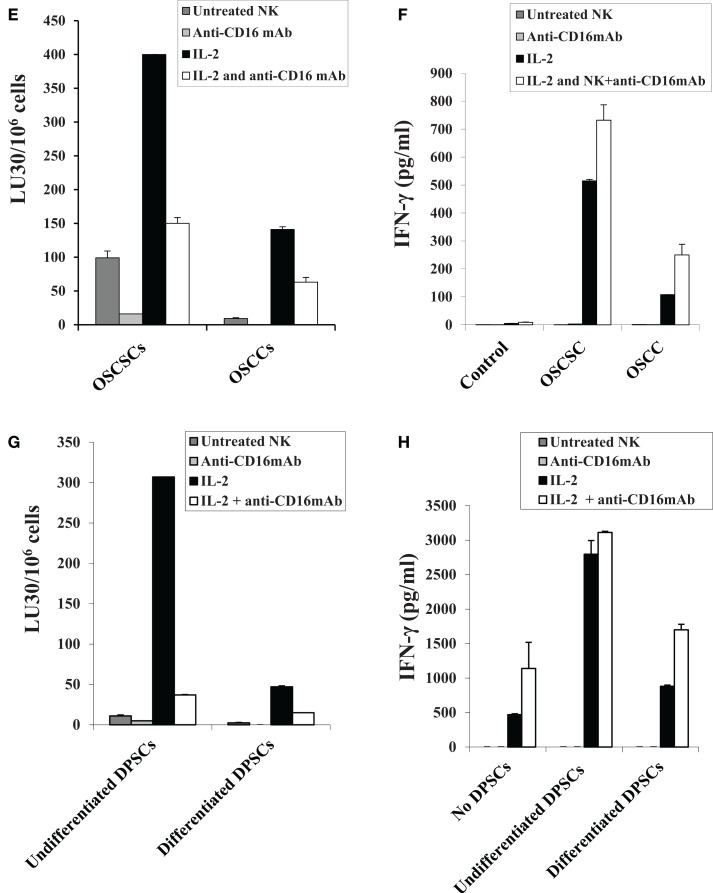
**Resistance of differentiated DPSCs and OSCCs but not stem-like OSCSCs or DPSCs to untreated, IL-2-treated, and IL-2 + anti-CD16-treated NK cell cytotoxicity; loss of NK cell cytotoxicity and gain in secretion of IFN-γ after NK cell receptor signaling**. OSCCs or OSCSCs were seeded at 1 × 10^5^ cells/well in 24-well plate for 24 h prior to the addition of highly purified NK cells pre-treated with IL-2 (1000 units/ml) for 24 h. NK cells were added to tumor cells at 2:1 effector to target ratio. At time 0 when NK cells were added to the tumor culture, a final concentration of 10 μg/ml of propidium iodide (PI) was also added. The cells were then subsequently tracked for over 72 h using time-lapse microscopy with Nikon Eclipse Ti-E inverted microscope fitted with a culture chamber to provide cells with a stable temperature of 37°C with 5% CO_2_. An image was taken every 15 min and a representation is shown at day 1, day 1½, and day 3. OSCSCs but not OSCCs which are lysed take up PI and appear orange in the time-lapse **(A)**. The surface expression of CD26, CD44, CD166, and CD326 on OSCCs and OSCSCs were assessed with flow cytometric analysis after staining with the respective PE-conjugated antibodies. Isotype control antibodies were used as control. The numbers on the right hand corner are the percentages and the mean channel fluorescence intensities for each histogram **(B)**. The surface expression of CD338 on OSCCs and OSCSCs was assessed by flow cytometric analysis after staining with PE-conjugated CD338 (right graphs in the histogram). Isotype control antibodies were used as control (left graphs in the histograms) **(C)**. OSCCs and OSCSCs were left untreated or treated with 10–80 μg/ml of cisplatin for 18 h, after which the tumor cells were washed with 1× PBS, detached, and stained with propidium iodide (PI) and percent cell death was determined using flow cytometric analysis **(D)**. NK cells were left untreated or treated with IL-2 (1000 units/ml), anti-CD16 mAb (3 μg/ml), or a combination of IL-2 (1000 units/ml) and anti-CD16 mAb (3 μg/ml) for 18 h before they were added to ^51^Cr-labeled OSCSCs and OSCCs **(E)** or undifferentiated and differentiated DPSCs **(G)**. NK cell-mediated cytotoxicity was determined using a standard 4 h ^51^Cr release assay and the lytic units 30/10^6^ cells were determined using inverse number of NK cells required to lyse 30% of the target cells × 100. NK cells were treated as described in **(E)** and each NK sample was either cultured in the absence or presence of OSCSCs and OSCCs **(F)** or undifferentiated and differentiated DPSCs **(H)** at an NK cell to target cell ratio of 0.5:1. After an overnight incubation, the supernatant was removed from the co-cultures and the levels of IFN-γ secretion were determined using specific ELISAs. One of minimum three representative experiments is shown in each of **(B–H)**.

NK cells were left untreated or treated with anti-CD16 antibody and/or IL-2 for 18–24 h before they were used with OSCSCs or their differentiated counterparts OSCCs. As shown previously and in here, using time-lapse microscopic analysis, NK cells mediated much higher lysis of stem-like OSCSCs when compared to differentiated OSCCs (Figure 1A). We had previously characterized stem-like OSCSCs and differentiated OSCCs based on their surface expression (29), and in this report we extended the characterization of stem-like OSCSCs to include other stem cell markers of CD26, CD326 (EpCam), CD166, and CD338 (Figures 1B,C). As shown in Figures 1B,C, OSCSCs expressed significantly higher amounts of CD26, CD326, and CD338 and lower amounts of CD166 similar to those reported previously (40–43). In agreement with the increased expression of CD338 (Figure 1C), OSCSCs were significantly more resistant to cisplatin (Figure 1D) and paclitaxel (manuscript in preparation) when compared to differentiated counterparts. In accordance to the data obtained with the time-lapse microscopy, both untreated and IL-2-treated NK cells mediated higher lysis of OSCSCs when compared to OSCCs in a 4-h ^51^Cr release assay (*P* < 0.05), and IL-2-treated NK cells secreted significant levels of IFN-γ in co-culture with OSCSCs when compared to OSCCs (Figures 1E,F). Anti-CD16 mAb treatment inhibited NK cell cytotoxicity against both OSCSCs and OSCCs (*P* < 0.05), however, it did not induce any appreciable secretion of IFN-γ (Figures 1E,F). The addition of the combination of IL-2 and anti-CD16 mAb treatment although inhibited NK cell cytotoxicity significantly against OSCSCs and OSCCs when compared to IL-2-activated NK cells alone (*P* < 0.05), it induced higher release of IFN-γ when cultured in the presence and absence of OSCSCs (Figures 1E,F). The levels of IFN-γ secretion remained much less in the co-cultures of IL-2 and/or anti-CD16 mAb-treated NK cells with OSCCs when compared to those cultured with OSCSCs (*P* < 0.05) correlating with the decreased cytotoxicity by IL-2-treated NK cells against OSCCs (Figures 1E,F). Therefore, anti-CD16 mAb in combination with IL-2 induced split anergy in NK cells resulting in a great loss of cytotoxicity but significant gain in secretion of IFN-γ against oral stem-like tumors (Figures 1E,F).

To determine whether similar results to those demonstrated above can be obtained by healthy untransformed primary stem cells and their differentiated counterparts, we performed the following experiments. NK cells were treated as described in Figure 1E before they were added to ^51^Cr-labeled DPSCs and their differentiated counterparts. As shown in Figure 1G, IL-2-treated NK cells mediated much higher lysis of undifferentiated DPSCs when compared to differentiated DPSCs (*P* < 0.05). Anti-CD16 mAb treatment inhibited NK cell cytotoxicity against both undifferentiated and differentiated DPSCs (*P* < 0.05), however, it did not induce any appreciable secretion of IFN-γ (Figure 1H). The addition of the combination of IL-2 and anti-CD16 mAb treatment although inhibited NK cell cytotoxicity against undifferentiated and differentiated DPSCs significantly, when compared to IL-2-activated NK cells alone (*P* < 0.05), it induced higher release of IFN-γ when cultured in the presence and absence of DPSCs (Figure 1H). The levels of IFN-γ secretion remained much less in the co-cultures of IL-2 or IL-2 and anti-CD16 mAb-treated NK cells with differentiated DPSCs when compared to those cultured with undifferentiated DPSCs (*P* < 0.05) correlating with the decreased cytotoxicity by IL-treated NK cells against differentiated DPSCs (Figures 1G,H). The levels of IFN-γ secretion in the co-cultures of undifferentiated DPSCs with IL-2 alone or IL-2 in combination with anti-CD16 mAb-treated NK cells plateaued (Figure 1H).

### Monocytes induce split anergy in NK cells resulting in a significant loss of NK cell cytotoxicity against OSCSCs and DPSCs and increased secretion of IFN-γ by the anergized NK cells

**Figure 2:**
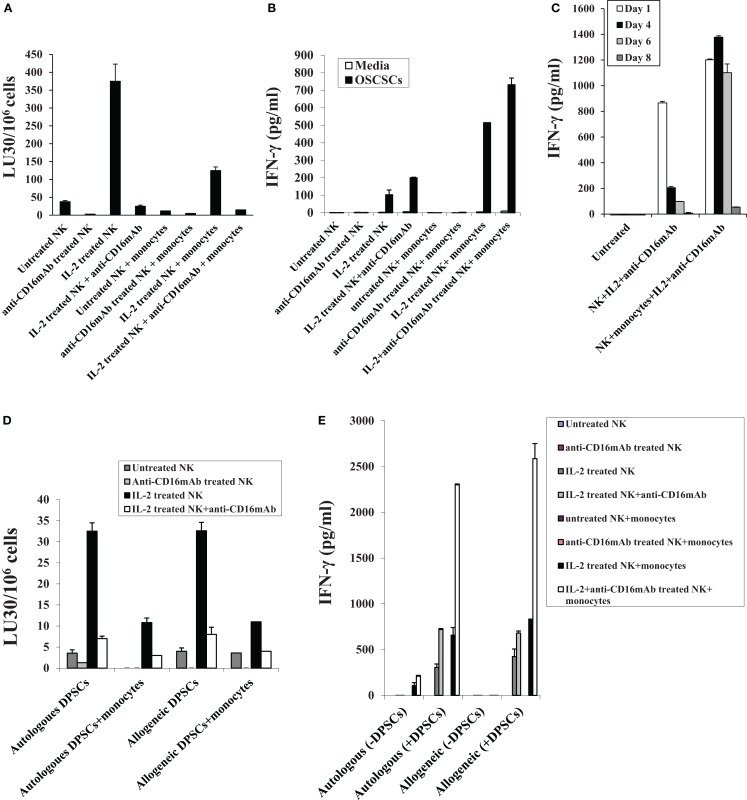
**Monocytes induce split anergy in NK cells resulting in a significant loss of NK cell cytotoxicity against OSCSCs and DPSCs and increased secretion of IFN-γ by the NK cells**. NK cells were purified from healthy donors and left untreated or treated with IL-2 (1000 units/ml), anti-CD16 mAb (3 μg/ml), or the combination of IL-2 (1000 units/ml) and anti-CD16 mAb (3 μg/ml) in the presence or absence of autologous monocytes (1:1 ratio of NK:monocytes) for 24 h, after which they were washed and added to ^51^Cr-labeled OSCSCs in a 4-h chromium release assay. Percent cytotoxicity was obtained at different effector to target ratio, and the lytic units 30/10^6^ cells were determined using inverse number of NK cells required to lyse 30% of the tumor cells × 100 **(A)**. NK cells were treated as described in Figure 2A and either cultured in the absence or presence of OSCSCs. After an overnight incubation, supernatants were removed from co-cultures and the levels of IFN-γ secretion were determined using specific ELISAs **(B)**. Highly purified NK cells were left untreated or treated as described in Figure 2A. After 1, 4, 6, and 8 days post-treatment, supernatants were removed from NK cell cultures and the levels of IFN-γ **(C)** release were determined using specific ELISAs. NK cells were purified from healthy donors and left untreated or treated with IL-2 (1000 units/ml), anti-CD16 mAb (3 μg/ml), or the combination of IL-2 (1000 units/ml) and anti-CD16 mAb (3 μg/ml) in the presence or absence of autologous and allogeneic monocytes (1:1 ratio of NK:monocytes) for 24 h, after which they were washed and added to ^51^Cr-labeled autologous and allogeneic DPSCs in a 4-h chromium release assay **(D)**. Percent cytotoxicity was obtained at different effector to target ratio, and the lytic units 30/10^6^ cells were determined using inverse number of NK cells required to lyse 30% of the tumor cells × 100. NK cells were treated as described in Figure 2D and either cultured in the absence or presence of autologous and allogeneic DPSCs (NK:DPSC of 0.2:1). After an overnight incubation, supernatants were removed from the co-cultures and the levels of IFN-γ secretion were determined using specific ELISAs **(E)**. One of minimum three representative experiments is shown in each of **(A–E)**.

Monocytes were purified from PBMCs and irradiated at 20 Gy. OSCSCs were co-cultured with allogeneic irradiated monocytes before they were labeled with ^51^Cr and used in the cytotoxicity assays against NK cells. NK cells were treated as indicated in Figure 1A and they were used in the cytotoxicity assays against OSCSCs and DPSCs. The addition of irradiated monocytes to OSCSCs significantly protected the OSCSCs (Figure 2A) from NK cell-mediated cytotoxicity (*P* < 0.05). Significant inhibition of NK cell cytotoxicity by monocytes could be observed against untreated and IL-2-treated NK samples (*P* < 0.05) (Figure 2A). Purified irradiated monocytes were also co-cultured with OSCSCs and untreated or IL-2 and/or anti-CD16 mAb pre-treated NK cells were added before the supernatants were removed and subjected to specific ELISAs for IFN-γ (Figure 2B). Irradiated monocytes cultured with OSCSCs increased secretion of IFN-γ substantially when added to IL-2 or IL-2 and anti-CD16 antibody-treated NK cells. Since monocytes alone or monocytes cultured with OSCSCs did not have any effect on IFN-γ secretion in all the experiments tested, we did not include them in Figure 2 (data not shown). In addition, when secretion of IFN-γ were determined in 8 days, IL-2 + anti-CD16 mAb-treated NK cells continuously secreted higher levels of IFN-γ only in the presence of monocytes at days 4 and 6, whereas IL-2 + anti-CD16 mAb-treated NK cells in the absence of monocytes had very low levels of secretion at days 4 and 6 (*P* < 0.05) (Figure 2C). No significant secretion could be observed in any of the samples at day 8 (Figure 2C). At day 1, IL-2 + anti-CD16 mAb-treated NK cells secreted high levels of IFN-γ and the levels increased in the presence of monocytes (Figure 2C). Monocytes in the absence of NK cells were unable to secrete IFN-γ (data not shown). Overall, these experiments indicated that monocytes induce split anergy in NK cells resulting in protection of OSCSCs from NK cell-mediated lysis while substantially increasing the secretion of IFN-γ.

Similar to OSCSCs, DPSCs were co-cultured with and without irradiated allogeneic and autologous monocytes before they were labeled with ^51^Cr and used in the cytotoxicity assays against allogeneic and autologous NK cells. NK cells were treated as indicated in Figure 1 before they were used in the cytotoxicity assays against DPSCs. As shown in Figure 2D, untreated, IL-2-treated, and IL-2 + anti-CD16 mAb-treated autologous NK cells lysed DPSCs to similar extents to those obtained with allogeneic NK cells. Addition of irradiated monocytes to DPSCs inhibited NK cell-mediated cytotoxicity in all treated NK samples and the levels of decrease were similar between autologous and allogeneic NK cells (Figure 2D). Significant inhibition of NK cell cytotoxicity could be obtained by treating autologous and allogeneic NK cells with IL-2 and anti-CD16 mAb when compared to IL-2-treated NK cells. In contrast, IL-2 and anti-CD16 mAb-treated NK cells secreted large amounts of IFN-γ and the levels substantially increased when added to monocyte-treated DPSCs (Figure 2E). Similar levels of IFN-γ secretion was obtained by both autologous and allogeneic NK cells when these cells were treated with IL-2 alone or IL-2 + anti-CD16 mAb in the presence and absence of DPSCs (Figure 2E).

### Supernatants from the combination of IL-2 and anti-CD16 mAb-treated NK cells induced resistance of OSCSCs and DPSCs to NK cell-mediated cytotoxicity and increased differentiation antigens on the surface of OSCSCs

**Figure 3:**
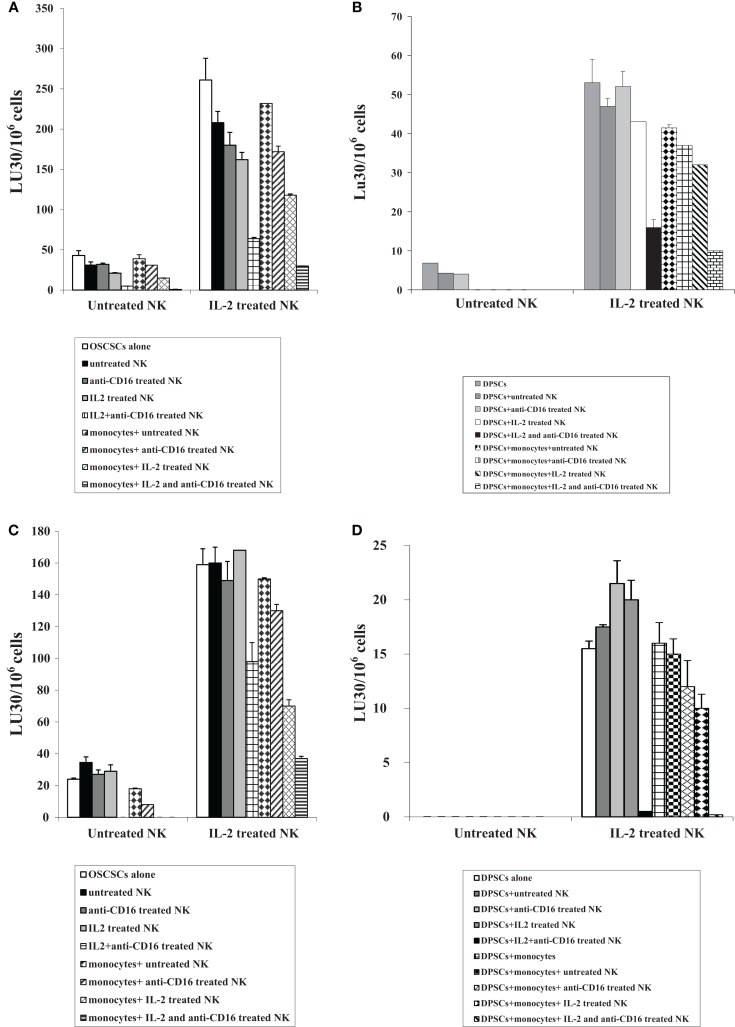
**Supernatants and paraformaldehyde-fixed NK cells treated with IL-2 in combination with anti-CD16 mAb with and without monocytes increased resistance of OSCSCs and DPSCs to NK cell-mediated cytotoxicity**. NK cells were purified from healthy donors and left untreated or treated with IL-2 (1000 units/ml), anti-CD16 mAb (3 μg/ml), or the combination of IL-2 (1000 units/ml) and anti-CD16 mAb (3 μg/ml) in the presence or absence of autologous monocytes (1:1 ratio of NK:monocytes) for 24 h, after which the same amounts of supernatants from different treatments of NK cells were removed and added to OSCSCs for a period of 5 days **(A)** or DPSCs for a period of 7 days **(B)**. NK supernatant-treated OSCSCs or DPSCs were then washed with 1× PBS, detached and labeled with ^51^Cr, and used in the cytotoxicity assay against freshly isolated NK cells. Untreated or IL-2-treated (1000 units/ml) NK cells were used to assess cytotoxicity against NK supernatant-treated target cells using a standard 4 h ^51^Cr release assay. Percent cytotoxicity was determined at different effector to target ratio, and the lytic units 30/10^6^ cells were determined using inverse number of NK cells required to lyse 30% of the tumor cells × 100. NK cells were purified from healthy donors and left untreated or treated with IL-2 (1000 units/ml), anti-CD16 mAb (3 μg/ml), or the combination of IL-2 (1000 units/ml) and anti-CD16 mAb (3 μg/ml) in the presence or absence of monocytes (1:1 ratio of NK:monocytes) for 24 h. Treated NK samples were then washed and fixed with 2% paraformaldehyde for 15 min before they were either added to OSCSCs at 0.75:1 for 5 days **(C)** or DPSCs at 2:1 for 7 days **(D)**. The fixed NK cells were then completely removed from OSCSCs and DPSCs and the target cell sensitivity to NK cell-mediated lysis were determined using a standard 4 h ^51^Cr release assay using freshly isolated and untreated or IL-2-treated (1000 units/ml) NK cells. Removal of fixed NK cells from stem cell cultures were assessed using microscopic observation. Percent cytotoxicity was determined at different effector to target ratio, and the lytic units 30/10^6^ cells were determined using inverse number of NK cells required to lyse 30% of the tumor cells × 100. One of minimum three representative experiments is shown in each of **(A–D)**.

NK cells were treated as described in Figure 1A before their supernatants were removed and added to OSCSCs as described in the Section “Materials and Methods.” Treatment of OSCSCs with IL-2 + anti-CD16 mAb-treated NK cell supernatants decreased NK cell-mediated cytotoxicity significantly by untreated and IL-2-treated NK cells (*P* < 0.05) (Figure 3A). Resistance of OSCSCs to NK cell-mediated cytotoxicity could also be observed after their treatment with IL-2 and much less with anti-CD16 mAb-treated NK supernatants, but the levels of NK resistance were significantly less when compared to those induced by IL-2 + anti-CD16 mAb-treated NK cell supernatants (*P* < 0.05) correlating with the degree of differentiation based on the surface receptors (please see below). Supernatants harvested from irradiated monocytes with IL-2 + anti-CD16-treated NK cells and added to OSCSCs resulted in a further increase in the resistance of OSCSCs to NK cell-mediated cytotoxicity (Figure 3A). Supernatants from irradiated monocytes and IL-2-treated NK cells were also able to induce resistance of OSCSCs (*P* < 0.05) but the levels of resistance were less when compared to that induced by supernatants obtained from the combination of IL-2 and anti-CD16 mAb-treated NK cells in the presence of irradiated monocytes (Figure 3A).

Similarly, treatment of DPSCs with IL-2 + anti-CD16 mAb-treated NK cell supernatants significantly decreased cytotoxicity mediated by IL-2-treated NK cells (*P* < 0.05) (Figure 3B). Addition of supernatants removed from the co-cultures of IL-2-treated NK cells with monocytes to DPSCs increased resistance of these cells against IL-2-treated NK cells, however, the highest resistance was observed in DPSCs treated with supernatants removed from cultures of monocytes with the combination of IL-2 and anti-CD16 mAb-treated NK cells (Figure 3B).

### Addition of paraformaldehyde-fixed IL-2 + anti-CD16 mAb-treated NK cells with and without irradiated monocytes to OSCSCs and autologous DPSCs-mediated resistance of OSCSCs and DPSCs against NK cell-mediated cytotoxicity

Similar to the treatment of OSCSCs with the NK cell supernatants, OSCSCs became resistant to NK cell-mediated cytotoxicity after their co-culture with IL-2 + anti-CD16 mAb-treated paraformaldehyde-fixed NK cells (*P* < 0.05) when compared to the addition of untreated or anti-CD16 mAb-treated or IL-2-treated fixed NK cells (Figure 3C). The complete removal of fixed NK cells from the OSCSCs prior to the cytotoxicity assays were determined by microscopic assessment. Co-cultures of IL-2 and IL-2 + anti-CD16 mAb-treated NK cells with monocytes before their fixation and addition to OSCSCs increased resistance of these cells against untreated and IL-2-treated NK cells and the highest resistance was observed in OSCSCs which were cultured with monocytes and the combination of IL-2 and anti-CD16 mAb-treated NK cells although monocytes cultured with IL-2-treated NK cells were also able to induce resistance in OSCSCs (Figure 3C).

Similarly, cultures of DPSCs with paraformaldehyde-fixed autologous IL-2 + anti-CD16 mAb pre-treated NK cells decreased NK cell-mediated cytotoxicity by untreated and IL-2-treated NK cells significantly (*P* < 0.05) (Figure 3D). Cultures of irradiated monocytes with the IL-2-treated NK cells before their addition to DPSCs increased resistance of these cells against IL-2-treated NK cells moderately, however, the highest resistance was observed in DPSCs which were cultured with monocytes and the combination of IL-2 and anti-CD16 mAb-treated NK cells (Figure 3D).

### Induction of NK resistance in OSCSCs correlated with the increased expression of CD54, B7H1, MHC class I, and decreased expression of CD44

**Figure 4:**
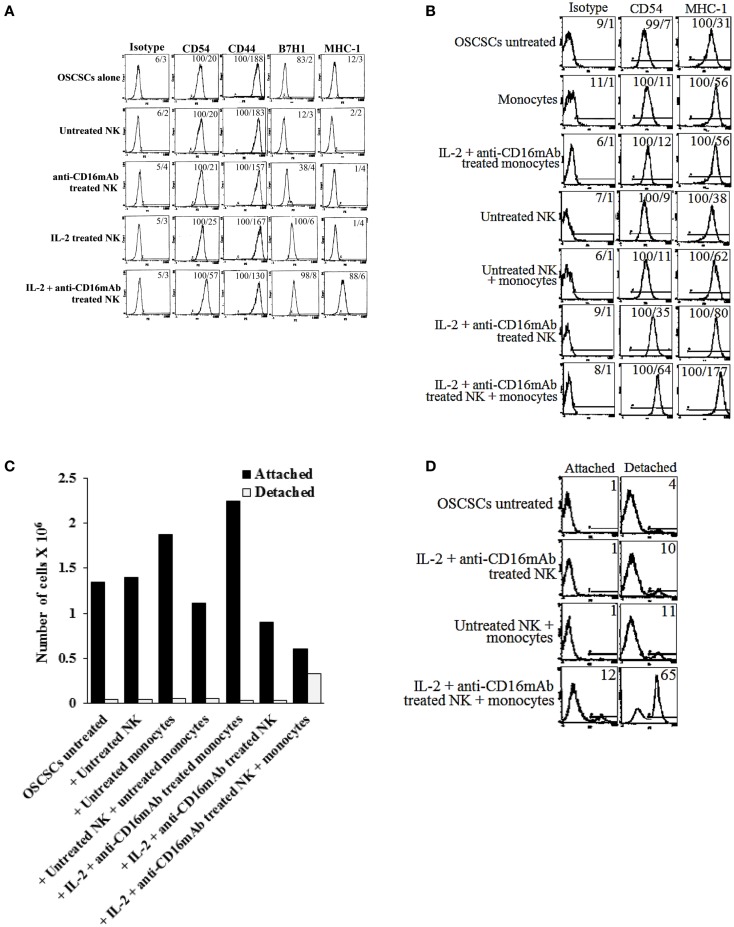
**Induction of resistance to NK cell-mediated cytotoxicity and inhibition of growth in OSCSCs correlated with the increased expression of CD54, B7H1, MHC class I, and decreased expression of CD44 on OSCSCs treated with supernatants from IL-2 + anti-CD16 mAb-treated NK cells with and without monocytes**. NK cells were purified from healthy donors and left untreated or treated with IL-2 (1000 units/ml), anti-CD16 mAb (3 μg/ml), or the combination of IL-2 (1000 units/ml) and anti-CD16 mAb (3 μg/ml) for 24 h. Thereafter, the same amounts of supernatants from different treatments of NK cells were removed and added to OSCSCs for 5 days. OSCSCs were then washed, and the expression of CD54, CD44, B7H1, and MHC class I were assessed after staining with the PE-conjugated antibodies using flow cytometry **(A)**. NK cells were purified from healthy donors and left untreated or treated with IL-2 (1000 units/ml), anti-CD16 mAb (3 μg/ml), or the combination of IL-2 (1000 units/ml) and anti-CD16 mAb (3 μg/ml) in the presence or absence of autologous monocytes (1:1 ratio of NK:monocytes) for 24 h. Thereafter, the same amounts of supernatants from different treatments of NK cells were removed and added to OSCSCs. After 5 days of incubation with the NK cell supernatants, OSCSCs were washed with 1× PBS, and the expression of CD54 and MHC-1 were assessed after staining with the PE-conjugated antibodies using flow cytometry **(B)**. Isotype control antibodies were used as controls. The numbers on the right hand corner are the percentages and the mean channel fluorescence intensities for each histogram. At the end of the incubation of OSCSCs with NK cell supernatants, OSCSCs which were remained attached to the plate and those which detached during the incubation period were collected separately, and the number of cells **(C)** and their viability **(D)** were assessed using microscopy and propidium iodide staining followed by flow cytometric analysis, respectively. One of minimum three representative experiments is shown in each of **(A–D)**.

We then compared NK cell resistance induced by the NK cell supernatant in OSCSCs to expression of key cell surface receptors. Among many surface receptors tested, CD44, CD54, B7H1, and MHC class I expression was found to correlate significantly with the differentiation and resistance of OSCSCs to NK cell-mediated cytotoxicity (Figure 4A). As shown in Figure 4A, the levels of CD54, MHC class I, and B7H1 increased substantially on OSCSCs whereas the levels of CD44 decreased in the presence of IL-2 + anti-CD16 mAb-treated NK cell supernatants (Figure 4A). Treatment of OSCSCs with IL-2 alone treated NK supernatants modulated the above mentioned surface receptors moderately, and the levels were less when compared to those modulated by the supernatants obtained from IL-2 + anti-CD16 mAb-treated NK cells (Figure 4A). As expected, supernatants obtained from the monocytes with IL-2 + anti-CD16 mAb-treated NK cells up-regulated both CD54 and MHC class I substantially more when compared to either monocytes alone or IL-2 + anti-CD16 mAb-treated NK cells in the absence of monocytes (Figure 4B). Since the highest increase in resistance to NK cell cytotoxicity and augmented expression of CD54, B7H1, and MHC class I were found in the presence of the treatment of OSCSCs with supernatants from IL-2 + anti-CD16 mAb-treated NK cells; i.e., anergized NK cells, we used this treatment hereafter for the remaining of the studies.

To determine growth dynamics of OSCSCs after treatment with the NK supernatants, the number of attached and detached OSCSCs were counted after treatment with the supernatants by microscopic evaluation and the levels of cell death were determined by staining with PI followed by flow cytometric analysis. As shown in Figure 4C, there was a decrease in the numbers of OSCSCs after their treatment with IL-2 and anti-CD16 mAb-treated NK cell supernatants when compared to untreated OSCSCs or those treated with untreated NK cell supernatants (Figure 4C). However, the highest decrease was observed when OSCSCs were cultured with the supernatants obtained from the combination of IL-2 + anti-CD16 mAb-treated NK cells with monocytes (Figure 4C). Interestingly, monocytes alone increased growth of the OSCSCs when compared to untreated OSCSCs (Figure 4C). When the numbers of detached OSCSCs were determined, no significant differences could be observed in those treated with NK supernatants alone and the highest increase was seen when OSCSCs were cultured with the supernatants obtained from the combination of IL-2 + anti-CD16 mAb-treated NK cells with monocytes (Figure 4C). Similarly, 12% increase in cell death could be seen when OSCSCs were treated with the combination of IL-2 + anti-CD16 mAb-treated NK cells with monocytes, whereas no cell death in attached OSCSCs could be observed when they were treated with the IL-2 + anti-CD16 mAb-treated NK cells in the absence of monocytes (Figure 4D). Although slight increases in cell death of detached OSCSCs were seen when these cells were treated with IL-2 + anti-CD16 mAb-treated NK cell supernatants (10%), the levels increased substantially in detached cells when OSCSCs were treated with the supernatants from the combination of IL-2 + anti-CD16 mAb-treated NK cells and monocytes (Figure 4D).

### Induction of resistance in OSCSCs to NK cell-mediated cytotoxicity by anergized NK cells is mediated by the combination of IFN-γ and TNF-α and not each cytokine alone

**Figure 5:**
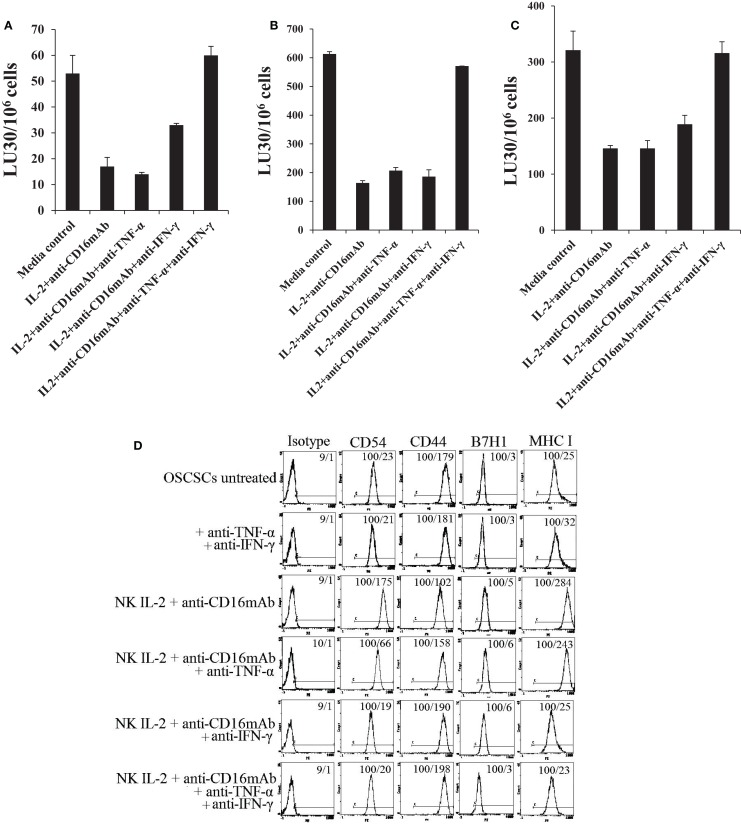

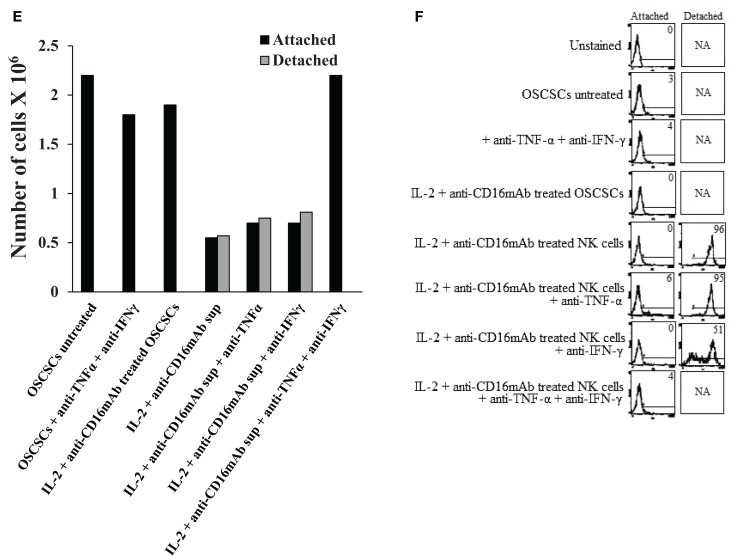
**Induction of resistance of OSCSCs to NK cell-mediated cytotoxicity and inhibition of growth in OSCSCs by IL-2 + anti-CD16 mAb-treated NK cells is mediated by the combination of IFN-γ and TNF-α and not each cytokine alone**. Highly purified NK cells were treated with the combination of IL-2 (1000 units/ml) and anti-CD16 mAb (3 μg/ml) for 24 h, after which supernatant was removed and added to OSCSCs in the presence and absence of anti-TNF-α (1:100) and/or anti-IFN-γ (1:100) or isotype control antibodies for a period of 5 days. The cytotoxicity against untreated and NK supernatant-treated OSCSCs in the presence of antibodies to untreated NK cells **(A)**, IL-2-treated NK cells **(B)**, or IL-2 and anti-CD16 mAb-treated NK cells **(C)** were assessed using a standard 4 h ^51^Cr release assay. Percent cytotoxicity was obtained at different effector to target ratio, and the lytic units 30/10^6^ cells were determined using inverse number of NK cells required to lyse 30% of the tumor cells × 100. The surface expression of CD54, CD44, B7H1, and MHC class I on untreated, anti-TNF-α (1:100) and anti-IFN-γ (1:100) in the absence of NK supernatant, IL-2 + anti-CD16 mAb treated NK supernatants in the presence and absence of anti-TNF-α and/or anti-IFN-γ as shown in the figure were assessed were assessed after PE-conjugated antibody staining using flow cytometric analysis. Isotype control antibodies were used as controls. The numbers on the right hand corner are the percentages and the mean channel fluorescence intensities in each histogram **(D)**. At the end of the incubation of OSCSCs with NK cell supernatants, OSCSCs which were remained attached to the plate and those which detached during the incubation period were collected separately, and the number of cells **(E)** and their viability **(F)** were assessed using microscopy, and propidium iodide staining followed by flow cytometric analysis, respectively. One of minimum three representative experiments is shown in each of **(A–F)**.

To examine the mechanisms by which OSCSCs become resistant by anergized NK cells, we determined NK cell-mediated cytotoxicity when OSCSCs were treated with supernatants of NK cells treated with anti-CD16 mAb and IL-2 in the presence and absence of each of IFN-γ and TNF-α antibodies alone or their combination. As shown in Figures 5A–C, the addition of each of the IFN-γ and TNF-α antibody alone had a slight inhibitory effect on the induction of resistance of OSCSCs by the supernatants of NK cells treated with IL-2 + anti-CD16 mAb, however, the combination of anti-IFN-γ and anti-TNF-α abrogated the resistance of treated OSCSCs completely (Figures 5A–C). The inhibition of OSCSCs resistance to NK cell-mediated cytotoxicity by the combination of anti-IFN-γ and anti-TNF-α antibodies could be observed when either untreated (Figure 5A), IL-2-treated (Figure 5B), or IL-2 + anti-CD16 mAb-treated NK cells (Figure 5C) were used to assess cytotoxicity. Addition of isotype control antibodies to IL-2 + anti-CD16 mAb-treated NK cell supernatants did not change the resistance of OSCSCs to NK cell-mediated cytotoxicity (Figure 5). Resistance of OSCSCs induced by supernatants from IL-2 + anti-CD16 mAb-treated NK cells correlated with increased expression of CD54, B7H1, and MHC class I as shown above and the addition of a combination of anti-TNF-α and anti-IFN-γ antibodies prevented the up-regulation of these receptors (Figure 5D). The effect of anti-IFN-γ mAb in the absence of anti-TNF-α antibody, however, was more dominant for surface receptor modulation than cytotoxicity or cell growth (please see below) since its addition abrogated the increase in surface receptor expression substantially (Figure 5D). In addition, the rate of OSCSC cell growth was decreased when supernatants obtained from IL-2 + anti-CD16-treated NK cells were added, and this decrease was completely inhibited in the presence of the combination of anti-IFN-γ and anti-TNF-α antibodies and not each antibody alone (Figure 5E). Moreover, when the numbers (Figure 5E) and viability (Figure 5F) of OSCSCs attached or detached from the culture plates were determined after the addition of supernatants from the IL-2 + anti-CD16 mAb-treated NK cells, decreased growth rate with equal numbers of attached and detached OSCSCs were seen and the addition of the combination of anti-TNF-α and anti-IFN-γ restored the growth rate and eliminated the increase in detached OSCSCs (Figures 5E,F). No or slight decreases in the viability of attached OSCSCs could be seen after their treatment with the supernatants from IL-2 + anti-CD16 mAb-treated NK cells. In contrast, significant increases in cell death of detached OSCSCs could be seen (Figure 5F). Interestingly, treatment of OSCSCs with supernatants from IL-2 + anti-CD16 mAb-treated NK cells in the presence of anti-IFN-γ alone demonstrated reduced death of detached OSCSCs (Figure 5F).

Similar results to those obtained with OSCSCs were also observed when undifferentiated or stem-like Mia-Paca-2 (MP2) pancreatic tumors or SCAP were used to treat with the supernatants of NK cells treated with IL-2 + anti-CD16 mAb in the presence and absence of anti-TNF-α and/or anti-IFN-γ (Figure S1 in Supplementary Material).

### Induction of differentiation and resistance to NK cell-mediated cytotoxicity of OSCSCs by anergized paraformaldehyde-fixed NK cells is mediated by the combination of IFN-γ and TNF-α and not each cytokine alone

**Figure 6:**
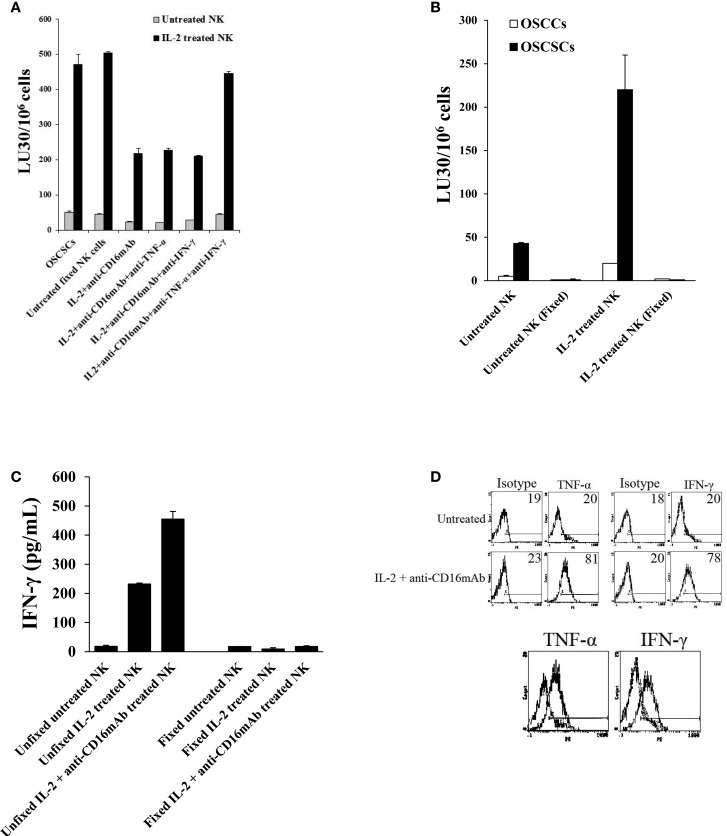

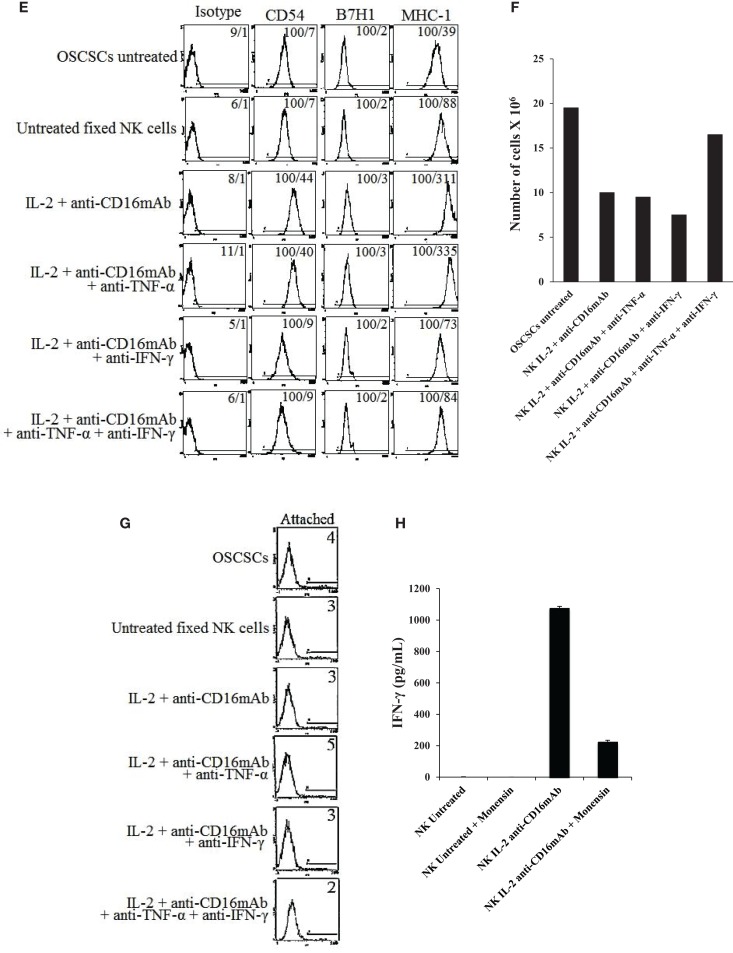

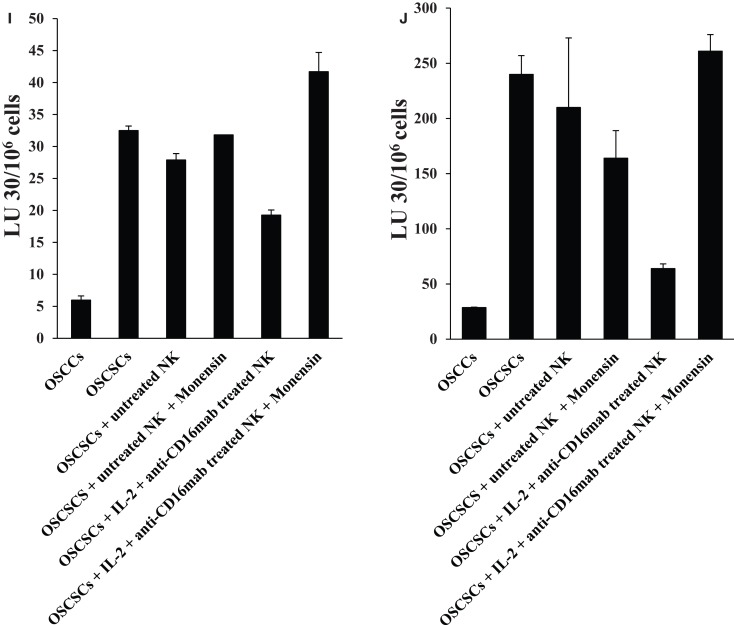

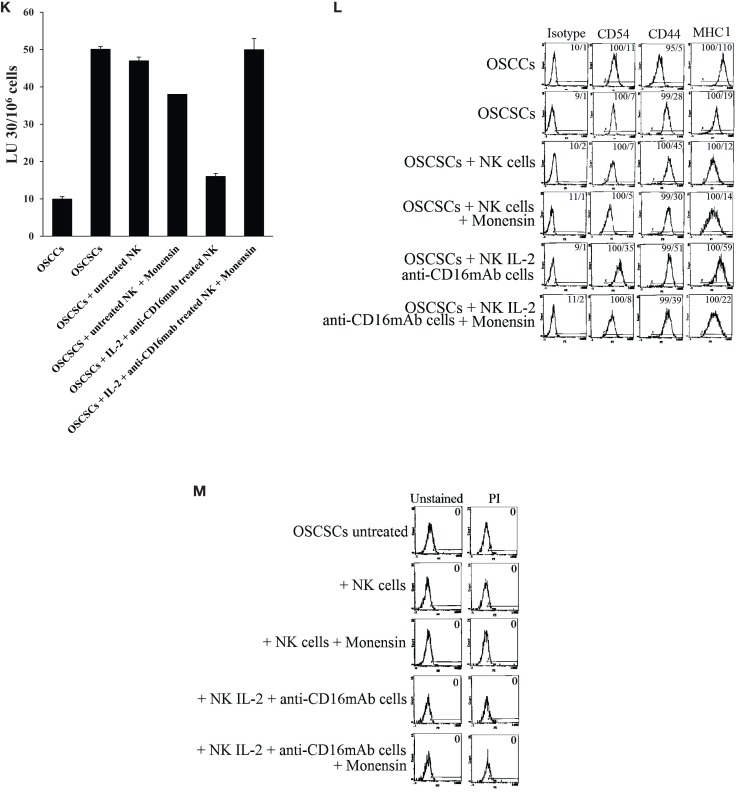
**Induction of differentiation, inhibition of cell growth, and resistance to NK cell-mediated cytotoxicity in OSCSCs induced by IL-2 + anti-CD16 mAb-treated and paraformaldehyde-fixed NK cells is mediated by the combination of IFN-γ and TNF-α and not each cytokine alone**. Highly purified NK cells were left untreated or treated with the combination of IL-2 (1000 units/ml) and anti-CD16 mAb (3 μg/ml) for 24 h, after which the NK cells were fixed with 2% paraformaldehyde and added to OSCSCs (0.75:1 NK:OSCSC ratio) in the presence and absence of anti-TNF-α (1:100) and/or anti-IFN-γ (1:100) or isotype control antibodies for a period of 5 days. The media containing the fixed NK cells were removed and extensively washed from each treated OSCSCs and the cytotoxicity against OSCSCs were assessed using freshly isolated untreated NK cells or those treated with IL-2 using a standard 4 h ^51^Cr release assay. The complete removal of fixed NK cells from OSCSCs was determined by microscopy. Percent cytotoxicity was obtained at different effector to target ratio, and the lytic units 30/10^6^ cells were determined using inverse number of NK cells required to lyse 30% of the tumor cells × 100 **(A)**. The lack of cytotoxic function of 2% paraformaldehyde-fixed untreated and IL-2-treated NK cells were assessed against OSCSCs and OSCCs. NK cells were left untreated or treated with IL-2 for 24 h before they were fixed with 2% paraformaldehyde **(B)**. NK cells were purified from healthy donor and left untreated or treated with IL-2 (1000 units/ml) and the combination of IL-2 (1000 units/ml) and anti-CD16 mAb (3 μg/ml). After an overnight treatment, NK cells were fixed with freshly prepared 2% paraformaldehyde and washed twice with 1× PBS. After 24 h post fixation, supernatants were then collected and the levels of IFN-γ were measured with specific ELISA **(C)**. NK cells purified from healthy donors were left untreated or treated with a combination of IL-2 (1000 units/ml) and anti-CD16 mAb (3 μg/ml) for 24 h, after which the NK cells were washed extensively and stained with PE-conjugated anti-TNF-α mAb followed by flow cytometric analysis. IFN-γ expression was assessed using purified mouse anti-human IFN-γ antibody followed by PE-conjugated goat anti-mouse IgG. Isotype control antibodies were used as controls. The numbers on the right hand corner are percentage positive for each histogram. The histogram overlay for the anti-TNF-α or anti-IFN-γ stained untreated (left) and IL-2 + anti-CD16 mAb-treated NK cells (right) are also shown in this figure. **(D)** The surface expression of CD54, CD44, B7H1, and MHC class I on untreated and fixed NK-treated OSCSCs as described above were assessed after PE-conjugated antibody staining followed by flow cytometric analysis. Isotype control antibodies were used as controls. The numbers on the right hand corner are the percentages and the mean channel fluorescence intensities for each histogram **(E)**. At the end of the incubation of OSCSCs with fixed NK cells, OSCSCs which were remained attached to the plate were collected, and the number of cells **(F)** and their viability **(G)** were assessed using microscopy, and propidium iodide staining followed by flow cytometric analysis, respectively. Highly purified NK cells were left untreated or treated with the combination of IL-2 (1000 units/ml) and anti-CD16 mAb (3 μg/ml) in the presence and absence of monensin (1:1300) for 24 h, after which each sample of NK cells were extensively washed and fixed with 2% paraformaldehyde and added to OSCSCs (0.75:1 NK:OSCSC) for a period of 5 days. Inhibition of IFN-γ release in monensin-treated NK cells before fixation with 2% paraformaldehyde were assessed by ELISA **(H)**. The results were compared to OSCSCs in the absence of fixed NK cells. The media containing the fixed NK cells were removed and extensively washed from each treated OSCSCs and the cytotoxicity against each condition of OSCSCs were assessed using freshly isolated untreated NK cells **(I)**, those treated with IL-2 **(J)** or treated with the combination of IL-2 + anti-CD16 mAb **(K)** using a standard 4 h ^51^Cr release assay. The complete removal of fixed NK cells from OSCSCs was determined by microscopy. Differentiated OSCCs were used as control for the differentiation of OSCSCs. Percent cytotoxicity was obtained at different effector to target ratio, and the lytic units 30/10^6^ cells were determined using inverse number of NK cells required to lyse 30% of the tumor cells × 100. The surface expression of CD54, CD44, and MHC class I on untreated and fixed NK-treated OSCSCs as described above were assessed after PE-conjugated antibody staining followed by flow cytometric analysis. Isotype control antibodies were used as controls. The numbers on the right hand corner are the percentages and the mean channel fluorescence intensities for each histogram **(L)**. At the end of the incubation of OSCSCs with fixed NK cells, the media containing the fixed NK samples were removed from OSCSCs and the viability of the cells were assessed using propidium iodide staining followed by flow cytometric analysis **(M)**. One of minimum three representative experiments is shown in each of **(A–M)**.

Similar to the data shown above, the addition of each of the IFN-γ and TNF-α antibody alone had a slight inhibitory effect on the resistance of OSCSCs induced by the paraformaldehyde-fixed NK cells treated with IL-2 + anti-CD16 mAb, however, the combination of anti-IFN-γ and anti-TNF-α abrogated the resistance of treated OSCSCs completely (Figure 6A). IL-2 and IL-2 + anti-CD16 mAb pre-treated and paraformaldehyde-fixed NK cells neither were able to mediate cytotoxicity (Figure 6B) nor secrete IFN-γ (Figure 6C) or TNF-α (data not show) after fixation, and they expressed membrane-bound TNF-α and IFN-γ when assessed by flow cytometric analysis using antibodies to TNF-α and IFN-γ (Figure 6D). The inhibition of OSCSCs resistance to NK cell-mediated cytotoxicity by the combination of anti-IFN-γ and anti-TNF-α antibodies could be observed when IL-2-treated NK cells were used to assess cytotoxicity (Figure 6A). Resistance of OSCSCs by IL-2 + anti-CD16 mAb-treated fixed NK cells correlated with increased expression of CD54, B7H1, and MHC class I and the addition of a combination of anti-TNF-α and anti-IFN-γ antibodies prevented up-regulation of these receptors (Figure 6E). Similarly, the effect of anti-IFN-γ mAb in the absence of anti-TNF-α antibody was more dominant for surface receptor expression than cytotoxicity or cell growth since its addition abrogated the increase in surface receptor expression substantially (Figures 6A,E,F). The rate of OSCSC cell growth was decreased when IL-2 + anti-CD16-treated fixed NK cells were added, and this decrease was completely inhibited in the presence of the combination of anti-IFN-γ and anti-TNF-α antibodies and not each antibody alone (Figure 6F). Moreover, when the numbers (Figure 6F) and viability (Figure 6G) of attached OSCSCs from the cultured plates were determined after the addition of IL-2 + anti-CD16 mAb-treated fixed NK cells, decreased growth rate of OSCSCs were seen and the addition of the combination of anti-TNF-α and anti-IFN-γ restored the growth rate (Figure 6F). No or slight decreases in the viability of attached OSCSCs could be seen after their treatment with the IL-2 + anti-CD16 mAb-treated fixed NK cells (Figure 6G). Although, increased numbers of detached OSCSCs could be observed in the presence of IL-2 + anti-CD16 mAb-treated fixed NK cells, accurate numbers of detached OSCSCs and their viability could not be established because of contamination with fixed NK cells.

Similar results to those obtained with OSCSCs were also observed when undifferentiated or stem-like MP2 or SCAP were used to treat with the IL-2 + anti-CD16 mAb-treated fixed NK cells in the presence and absence of anti-TNF-α and/or anti-IFN-γ (data not shown).

To determine whether monensin-treated fixed anergized NK cells lose the ability to induce resistance of OSCSCs against NK cell-mediated cytotoxicity, first we established that treatment of NK cells with IL-2 + anti-CD16 mAb in the presence of monensin blocked substantially the release of IFN-γ in the supernatants (Figure 6H). Addition of monensin in combination with IL-2 + anti-CD16-treated and fixed NK cells to OSCSCs inhibited resistance of OSCSC against untreated (Figure 6I), IL-2 (Figure 6J), or IL-2 + anti-CD16 mAb-treated NK cells (Figure 6K) when compared to IL-2 + anti-CD16 mAb-treated fixed NK cells in the absence of monensin treatment. Treatment of NK cells with the combination of IL-2 + anti-CD16 mAb and monensin inhibited the increase in CD54 and MHC class I expression on OSCSCs when compared to those induced by IL-2 + anti-CD16 mAb-treated and fixed NK cells (Figure 6L). No cell death could be observed in attached OSCSCs with any of treatments (Figure 6M).

### Combination of rTNF-α and rIFN-γ induce differentiation and resistance of OSCSCs to NK cell-mediated cytotoxicity

**Figure 7:**
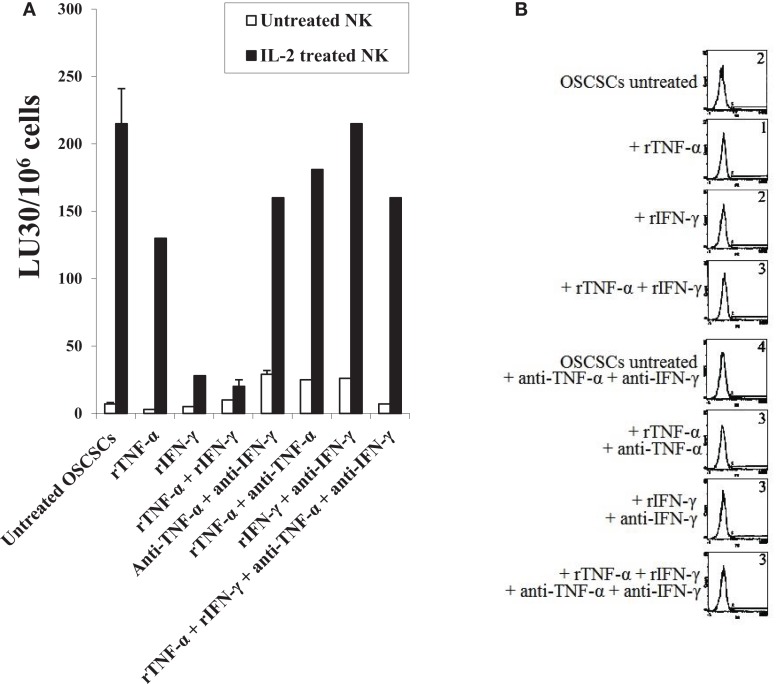

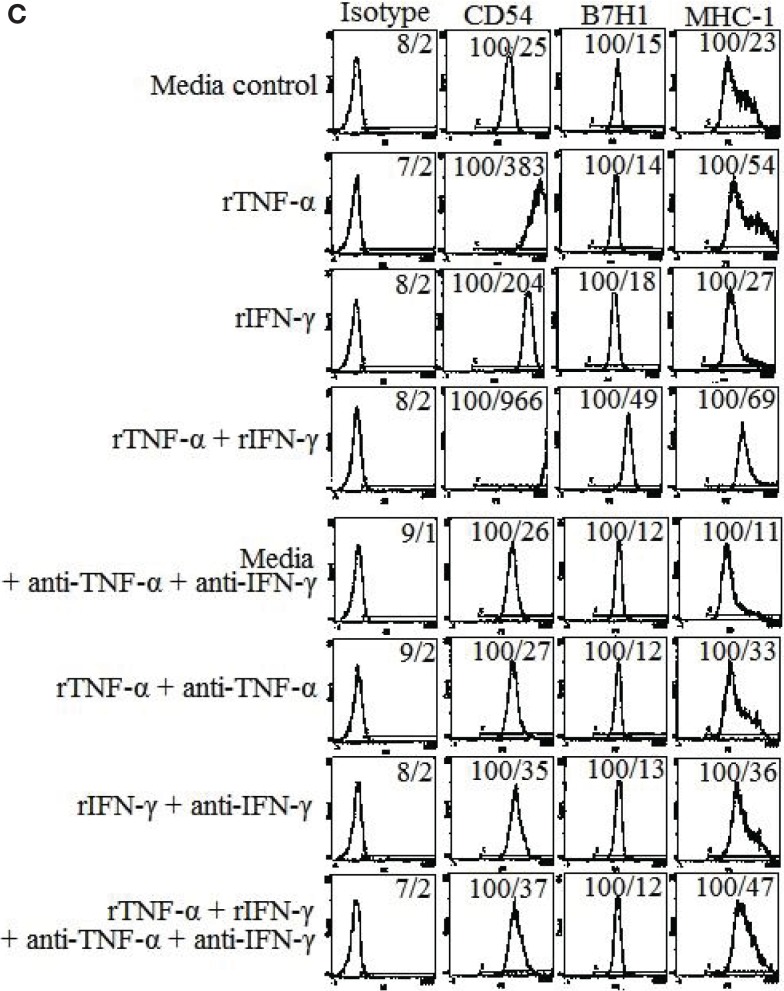
**Combination of rTNF-α and rIFN-γ induce differentiation and resistance of OSCSCs and SCAP to NK cell-mediated cytotoxicity**. OSCSCs **(A)** were left untreated or treated with recombinant human TNF-α (2 ng/ml), recombinant human IFN-γ (5 units/mL), or the combination of human TNF-α (2 ng/ml) and recombinant human IFN-γ (5 units/ml) in the presence or absence of antibodies against TNF-α (1:100) and/or IFN-γ (1:100) for 24 h. Afterwards, the cells were detached from the tissue culture plates and labeled with ^51^Cr and used in a standard 4 h chromium release assay against untreated and IL-2-treated (1000 units/ml) NK cells. Pre-treatment of NK cells with IL-2 were carried out for 18–24 h. Percent cytotoxicity was determined at different effector to target ratio, and the lytic units 30/10^6^ cells were determined using inverse number of NK cells required to lyse 30% of the tumor cells ×100. OSCSCs were treated as described in Figure 2A and their viability was assessed using propidium iodide staining followed by the flow cytometric analysis **(B)**. Surface expressions of CD54, CD44, B7H1, and MHC-1 on OSCSCs **(C)** treated as described in Figure 2A were determined using staining with PE-conjugated antibodies followed by flow cytometric analysis. Isotype control antibodies were used as controls. The numbers on the right hand corner are the percentages and the mean channel fluorescence intensities in each histogram. One of minimum three representative experiments is shown in each of **(A–C)**.

Oral squamous cancer stem cells were treated with rTNF-α and/or rIFN-γ in the presence and absence of anti-TNF-α mAb or anti-IFN-γ antiserum. As shown in Figure 7A, addition of rTNF-α to OSCSCs was able to induce some resistance against IL-2-treated NK cell-mediated cytotoxicity and this resistance was moderately blocked by the addition of anti-TNF-α antibody. rIFN-γ-treated OSCSCs were significantly more resistant to IL-2-treated NK cells when compared to rTNF-α-treated OSCSCs and the addition of anti-IFN-γ blocked resistance (*P* < 0.05) (Figure 7A). Since the addition of rIFN-γ had reduced the NK cytotoxicity substantially, further decrease with the combination of rTNF-α and rIFN-γ was difficult to demonstrate in this experiment (Figure 7A). No significant cell death could be observed in all treated samples at the concentrations of rTNF-α and rIFN-γ tested (Figure 7B). However, at the higher concentration of rTNF-α and rIFN-γ, when the combination of rTNF-α and rIFN-γ were used, an increase in the numbers of detached OSCSCs and cell death in both the attached and detached OSCSCs but not MP2 or SCAP could be observed (data not shown). Both rTNF-α and rIFN-γ were able to upregulate CD54 and MHC class I expression significantly, however, only rIFN-γ was able to upregulate B7H1 expression moderately on OSCSCs (Figure 7C). Addition of a combination of rTNF-α and rIFN-γ synergistically induced the expression of CD54 and B7H1, but not MHC class I (Figure 7C). Similar results to those obtained with OSCSCs were also obtained with SCAP and DPSCs. RTNF-α induced resistance to NK cell-mediated cytotoxicity less than rIFN-γ and the combination of rIFN-γ and rTNF-α further reduced IL-2-mediated cytotoxicity (Figure S2A in Supplementary Material). Unexpectedly, the level of CD44 was augmented by the treatment of rTNF-α and rIFN-γ and their combination, and the addition of anti-TNF-α and anti-IFN-γ antibodies blocked the increase substantially on DPSCs whereas it inhibited the increase in CD54, B7H1, and MHC class I expression by the combination of rTNF-α and rIFN-γ (Figure S2B in Supplementary Material).

### Antibodies to CD54 or LFA-1 was unable to inhibit differentiation in OSCSCs

**Figure 8:**
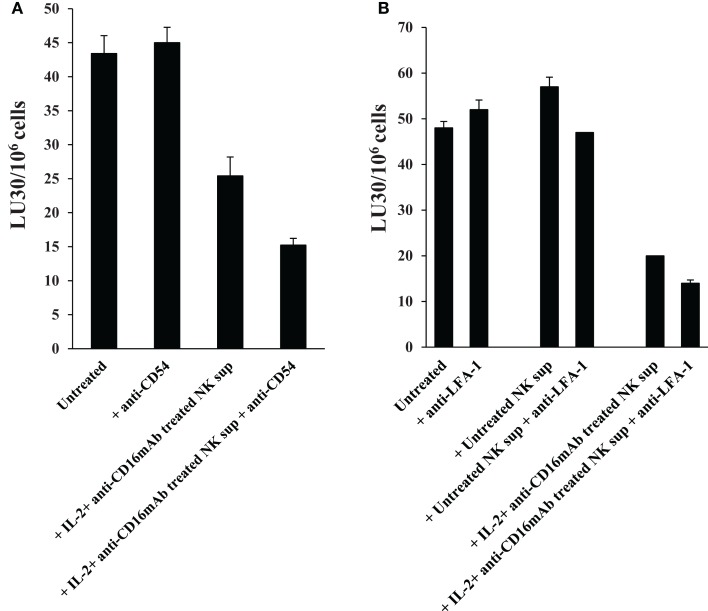
**Antibodies to CD54 or LFA-1 was unable to reverse differentiation but inhibited moderately the cytotoxicity of NKs against differentiated OSCSCs**. Highly purified NK cells were left untreated or treated with the combination of IL-2 (1000 units/ml) and anti-CD16 mAb (3 μg/ml) for 24 h, after which the same amounts of supernatants were removed and added to the OSCSCs in the presence and absence of anti-CD54 mAb (10 μg/ml) **(A)** and anti-LFA-1 (1:100) **(B)** for 5 days. Treated OSCSCs were then washed extensively and used in a standard 4 h ^51^Cr release assay against IL-2-activated (1000 units/ml) NK cells. Pre-treatment of NK cells with IL-2 were carried out for 18–24 h. Percent cytotoxicity was determined at different effector to target ratio, and the lytic units 30/10^6^ cells were determined using inverse number of NK cells required to lyse 30% of the tumor cells ×100.

Addition of antibodies to CD54 (Figure 8A) or its ligand LFA-1 (Figure 8B) in the initiation of differentiation of OSCSCs with supernatants from IL-2 + anti-CD16 mAb-treated NK cells for the duration of differentiation and their subsequent removal by extensive washing before labeling of OSCSCs with ^51^Cr and addition to freshly isolated IL-2-treated NK cells did not reverse differentiation in supernatant differentiated OSCSCs, however, it did inhibit cytotoxicity moderately in IL-2 + anti-CD16 mAb supernatant differentiated OSCSCs. In addition, unlike the effect of antibodies to TNF-α and IFN-γ, addition of anti-CD54 or anti-LFA-1 did not restore the numbers of OSCSCs to the levels seen with the untreated OSCSCs as seen in Figure 5E (data not shown). Moreover, the addition of CD54 and LFA-1 antibodies to untreated OSCSCs and those treated with untreated NK supernatants for the duration of differentiation and extensive washing to remove remaining antibodies before ^51^Cr labeling and culture with freshly isolated NK cells in ^51^Cr release assay did not change NK cell cytotoxicity (Figures 8A,B).

### Antibodies to B7H1 and MHC class I did not change or increased cytotoxicity of OSCSCs differentiated by IL-2 + anti-CD16 mAb-treated NK supernatants respectively

**Figure 9:**
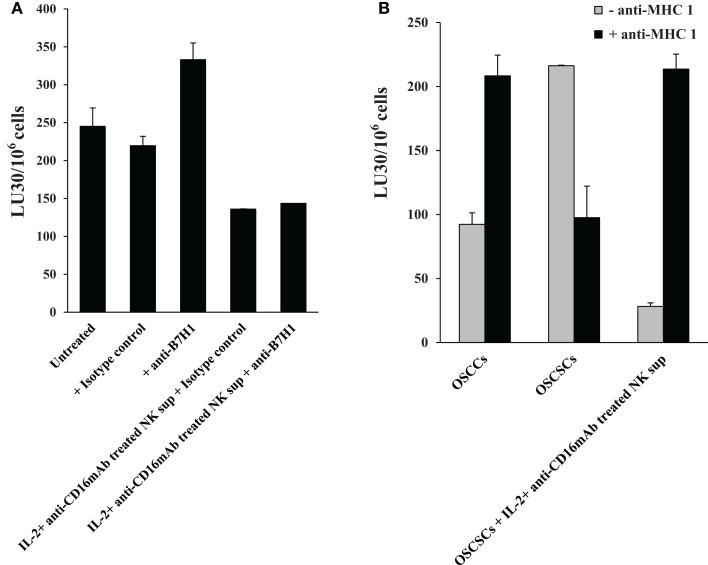
**Antibodies to MHC class I increased cytotoxicity of OSCSCs differentiated by IL-2 + anti-CD16 mAb-treated NK supernatants, whereas antibodies to B7H1 did not change cytotoxicity**. OSCSCs were treated with supernatants harvested from untreated or IL-2 + anti-CD16 mAb-treated NK cells for 5 days. Treatment of NK cells with the combination of IL-2 + anti-CD16 mAb were carried out for 18–24 h before the supernatants were removed and added to OSCSCs. Thereafter, treated OSCSCs were washed and labeled with ^51^Cr and used in the cytotoxicity assay in the presence and absence of anti-B7H1 (5 μg/ml) before their addition to NK cells. Percent cytotoxicity was determined at different effector to target ratio, and the lytic units 30/10^6^ cells were determined using inverse number of NK cells required to lyse 30% of the tumor cells ×100 **(A)**. OSCSCs were left untreated or treated with the supernatants prepared from the IL-2 + anti-CD16 mAb-treated NK cells for 5 days before they were washed, ^51^Cr-labeled, and treated with either isotype control antibody or anti-MHC class I antibody (1:100) for 10 min prior to their use in the cytotoxicity assay against IL-2-treated (1000 units/ml) NK cells. OSCCs were also ^51^Cr-labeled and treated with and without isotype control or anti-MHC class I (1:100) antibodies before they were used in the cytotoxicity assay against IL-2-treated NK cells. To prepare NK cell supernatant for the treatment of OSCSCs, NK cells were treated with the combination of IL-2 (1000 units/ml) and anti-CD16 mAb (3 μg/ml) for 18–24 h before the supernatants were harvested and added to OSCSCs. The treatment of IL-2 (1000 units/ml) was also carried out for 18–24 h before their use in the cytotoxicity assay. Percent cytotoxicity was determined at different effector to target ratio, and the lytic units 30/10^6^ cells were determined using inverse number of NK cells required to lyse 30% of the tumor cells ×100 **(B)**.

In contrast to anti-CD54 or anti-LFA-1, which were added at the initiation of differentiation and were present for the entire differentiation period, antibodies to B7H1 and MHC class I were added after the differentiation in the cultures of NK cells with the ^51^Cr-labeled differentiated targets only for 4 h to assess their significance in cytotoxicity and not differentiation since B7H1 and MHC class I were shown previously to regulate cytotoxicity of NK cells and were found to be elevated on the surface of OSCSCs differentiated with the IL-2 + anti-CD16 mAb-treated NK supernatants. As shown in Figure 9A, the addition of B7H1 antibody even though moderately increased cytotoxicity when added to untreated OSCSCs, it had no effect when added to IL-2 + anti-CD16 mAb NK supernatant differentiated OSCSCs even though the surface expression of B7H1 was significantly elevated on differentiated OSCSCs. In contrast, the addition of anti-MHC class I significantly increased cytotoxicity when added to IL-2 + anti-CD16 mAb NK supernatant differentiated OSCSCs, whereas it had inhibitory effect when added to untreated OSCSCs (Figure 9B). Addition of anti-MHC class I to well-differentiated OSCCs also increased cytotoxicity significantly (Figure 9B).

## Discussion

In this paper, we provide evidence that conditioned or anergized NK cells have the ability to induce resistance and differentiation of stem cells through secreted factors and direct cell–cell contact. Both secreted and membrane-bound IFN-γ and TNF-α were found to be important for the induction of resistance of OSCSCs and DPSCs to NK cell-mediated cytotoxicity, and inhibition of cell growth and proliferation, since combination of antibodies to TNF-α and IFN-γ and not each one alone was able to restore susceptibility to NK-mediated cytotoxicity and increase the number of tumor cells. On the other hand, IFN-γ appears to be more dominant in increasing cell surface receptors on differentiated OSCSCs and DPSCs since the addition of anti-IFN-γ antibody was able to block the expression of CD54, MHC class I, and B7H1 largely, even though the addition of the combination of rIFN-γ and rTNF-α was found to induce CD54, B7H1, and MHC class I expression synergistically. IL-2 and anti-CD16 mAb-activated fixed NK cells expressed membrane-bound TNF-α and IFN-γ and mediated differentiation and resistance of OSCSCs and DPSCs. Indeed, both soluble and immobilized IFN-γ was shown to mediate neuronal differentiation in a dose-dependent manner (44), and membrane-bound IFN-γ has previously been shown to protect mice from metastasis of Lewis lung carcinomas to a comparable extent to that mediated by the secreted cytokines (45). In addition, membrane-bound TNF-α confers protection against mycobacterial infection (46). These studies underscore the significance of both secreted and membrane-bound forms of TNF-α and IFN-γ in tumor therapy and infections. Moreover, the addition of monensin to IL-2 + anti-CD16 mAb pre-treated fixed NK cells was also able to prevent differentiation, and increase the numbers, viability, and susceptibility of stem cells to NK cell-mediated cytotoxicity in the presence of lower expression of CD54, MHC class I, and B7H1 surface receptors. These studies indicated that both membrane-bound as well as secreted forms of TNF-α and IFN-γ are important in differentiation of stem cells.

When the extent of resistance to NK cell-mediated cytotoxicity, cell growth, attachment to culture plates, and viability were determined in OSCSCs, MP2, and DPSCs after differentiation with the supernatants or fixed NK cells treated with IL-2 + anti-CD16 mAb, distinct cell signatures in respect to the outcome of differentiation were obtained for each cell type. In addition, the amount of supernatants used, and the length of treatment time were two important factors influencing the outcome of differentiation. While OSCSCs were sensitive to the differentiation effect of NK cell supernatants and exhibited detachment and cell death at relatively lower amounts of supernatant treatment, and in shorter period of treatment time, MP2, SCAP, and DPSCs were significantly more resistant, and they neither detached nor underwent cell death in the detached population at higher amounts of NK supernatants and in longer period of treatment. These differences could be due to the levels of TNF-α and IFN-γ receptor expression on each cell type and is the subject of our future studies. In addition, such differences may be the basis for why certain tumors are better controlled by the NK cells than the others, and provide the rationale for designing effective strategies to eliminate tumors. In agreement, differential effects of IFN-γ and TNF-α in either causing resistance in tumors or sensitizing them to NK cell-mediated cytotoxicity has also been reported previously (47–49).

Since CD54 was strongly elevated during differentiation, we aimed at understanding its role in differentiation. Addition of anti-CD54 or anti-LFA-1 antibodies at the initiation of differentiation of OSCSCs with IL-2 + anti-CD16 mAb-treated NK supernatants, did not change differentiation of OSCSCs since no restoration of cell number could be observed (data not shown), and their addition were moderately inhibitory for the cytotoxicity of NK cells in IL-2 + anti-CD16 mAb supernatant differentiated OSCSCs suggesting that cell contact through CD54 and LFA-1 may not be important for the differentiation of OSCSCs by the NK supernatants. In addition, both B7H1 and MHC class I are shown to regulate cytotoxicity of NK cells and are elevated on the surface of NK differentiated OSCSCs. Addition of antibodies to B7H1 did not increase cytotoxicity of NK cells against differentiated OSCSCs even though the surface expression of B7H1 was significantly elevated on differentiated OSCSCs. In contrast, addition of antibody to MHC class I significantly elevated cytotoxicity of NK cells against NK differentiated OSCSCs and this correlated with significant augmentation of MHC class I on NK differentiated OSCSCs. Similarly, the addition of anti-MHC class I antibody to the co-cultures of NK cells with differentiated primary OSCCs, which naturally express high surface MHC class I expression, mediated increased cytotoxicity by the NK cells. In contrast, addition of antibodies to MHC class I in the co-cultures of NK cells with stem-like OSCSCs was inhibitory for NK cell cytotoxicity. This inhibition could be due to the effect of anti-MHC class I antibody on NK cells and not on the tumor cells since we have previously shown that the addition of anti-MHC class I antibody to CD16 mAb-activated NK cells mediated significant inhibition of NK cell cytotoxicity (25). Whether increase in NK cell cytotoxicity against differentiated tumors by anti-MHC class I is due to ADCC or blocking of inhibitory ligands on NK cells awaits future investigation.

It is likely that other immune effectors such as T cells and NKT cells may also contribute to the pool of IFN-γ and TNF-α when they are activated, and thus they may be important for driving differentiation of tumors. In this regard, CD4+ Th1 cells may play a crucial role. Future studies should be able to establish the significance of other cell types in differentiation and resistance of tumor cells.

**Figure 10:**
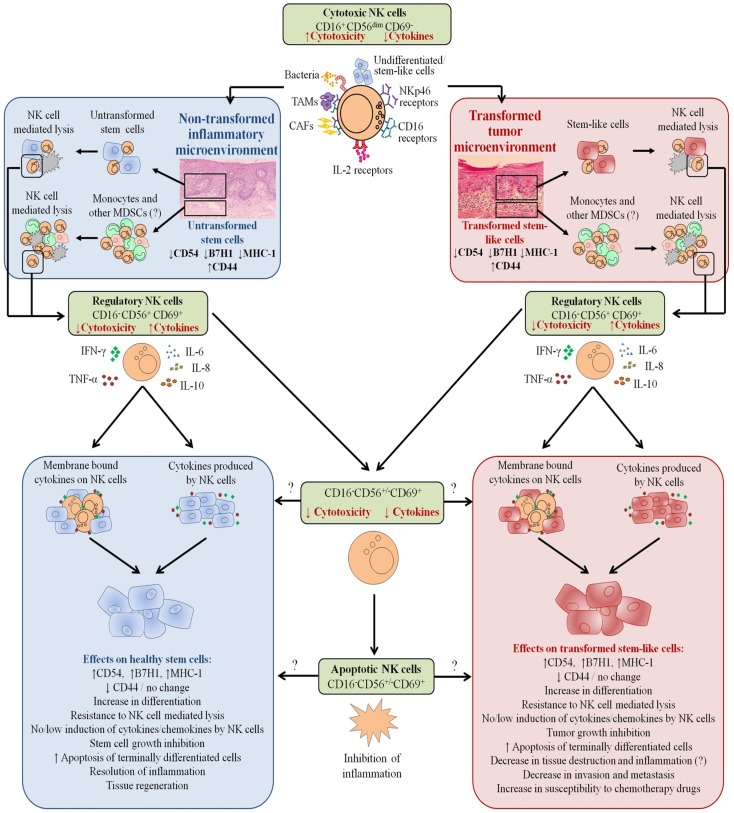
**Hypothetical model of induction of anergized NK cells by immune inflammatory cells and by the effectors of connective tissue to support differentiation of non-transformed stem cells and cancer stem cells resulting in their resistance to NK cell-mediated cytotoxicity**. NK cell anergy in tumor microenvironment, as well as in non-transformed immune inflammatory microenvironment, is shown. Significant infiltration of immune effectors right beneath the epithelial layer can be seen in a connective tissue area where immune inflammatory cells are likely to anergize NK cells to lose cytotoxicity and gain the ability to secrete cytokines, a term which we previously coined “split anergy” in NK cells, and to support differentiation of the basal epithelial layer containing stem cells. NK cells are likely to encounter and interact with other immune effectors such as monocytes/macrophages or other myeloid-derived suppressor cells (MDSCs), or with connective tissue-associated fibroblasts (CAF), in order to be conditioned to form anergized/regulatory NK (NKreg) cells. NK cells may also directly interact with stem cells at the base of the epithelial layer, in which case by eliminating their bound stem cells, they can become conditioned to support differentiation of other stem cells. NK cell-differentiated epithelial cells will no longer be killed or induce cytokine secretion by the NK cells, resulting in the resolution of inflammation.

Our results suggest two very important functions for the NK cells. One potential function of NK cells is to limit the number of stem cells, and second to support differentiation of the stem cells and their progeny. In respect to the oral squamous cell carcinomas since the majority of immune effectors are found at the connective tissue area (Figure 10) (34, 36–38, 50), it is likely that NK cells may first encounter and interact with either the other immune effectors or the effectors of connective tissue such as fibroblasts. However, there is also the possibility that NK cells may first encounter the basal epithelial stem cells in which case by eliminating the stem cells, they too can become anergized (Figure 10). By eliminating a subset of stem cells or after their interaction with other immune inflammatory cells or effectors of connective tissue, NK cells could then be in a position to support differentiation of remaining stem cells since they will be conditioned to lose cytotoxicity and induce cytokine and growth factor secretion. It is interesting to note that all of the immune effectors isolated from oral gingival tissues of healthy as well as diseased gingiva have CD69+ phenotype, with the exception that the numbers of immune effectors are much less in the healthy oral gingival tissues when compared to diseased tissues (34, 36–38, 50) (manuscript in preparation). In addition, lack of significant infiltration of NK cells in the tumor nest and the localization of NK cells in the immune-rich compartment, which surrounds the tumor in other tumor types, also provides the rationale and the means for the induction of split anergy in NK cells primarily by other immune effectors in the tumor microenvironment (50). Such mechanisms of NK cell conditioning by the other immune effectors such as myeloid-derived suppressor cells (MDSCs) explain why the cytotoxic function of NK cells are greatly reduced in the tumor microenvironment as well as in circulating NK cells.

There should be two distinct strategies to eliminate tumors, one which targets stem cells and the other which targets differentiated cells. Since cancer stem cells are found to be more resistant to chemotherapeutic drugs but sensitive to NK cell-mediated killing while differentiated oral tumors are more resistant to NK cell-mediated killing but relatively more sensitive to chemotherapeutic drugs (Figure 1), combination therapy should be considered for the elimination of both undifferentiated and differentiated tumors. In addition, since a great majority of patient NK cells have modified their phenotype to support differentiation of the cells, they may not be effective in eliminating cancer stem cells. Therefore, these patients may benefit from repeated allogeneic NK cell transplantation for elimination of cancer stem cells. In this regard, depletion of NK anergizing effectors such as monocytes in the tumor microenvironment which condition NK cells to lose cytotoxicity, via radiation or chemotherapeutic drugs before allogeneic NK cell transplantation should in theory provide such strategy for targeting the cancer stem cells by the NK cells. However, this strategy may also halt or decrease the ability of NK cells to drive optimal differentiation of the tumors and tilt the balance toward a more inflammatory tumor microenvironment which may run the risk of fueling the growth and expansion of more cancer stem cells. Indeed, NK supernatant differentiated tumors were unable to trigger secretion of cytokines and chemokines, providing a less inflammatory tumor microenvironment which may limit the growth and expansion of tumor cells (manuscript submitted).

Alternatively, a strong tumor differentiating microenvironment may be induced by the balanced release and membrane expression of key cytokines by the NK cells anergized with effectors such as monocytes or healthy stem cells in hope that most if not all of the newly arising cancer stem cells are induced to differentiate in order to become less aggressive. The benefit of such approach will be the ability of chemotherapeutic drugs to target the differentiated tumors in addition to the lack of differentiated tumors to metastasize (data not shown). Indeed, our recent *in vivo* data indicated that cancer stem cells have the ability to grow faster and metastasize, whereas the differentiated tumors grew slower and remained localized for a long period of time without metastasizing (manuscript in preparation). It is possible that the successful cancer therapy may lie between a balance in the two abovementioned approaches depending on the type of the tumor and the status of patients’ immune system. The most dangerous and devastating outcome of the cancer is its ability to deplete NK cells and other immune inflammatory cells. In this case, not only cancer stem cells will be surviving but they will also remain poorly differentiated which may establish a vicious cycle of tumor growth and loss of immune effectors in the tumor microenvironment and in the periphery. NK cell immunotherapy in these patients should be highly beneficial.

## Conflict of Interest Statement

The authors declare that the research was conducted in the absence of any commercial or financial relationships that could be construed as a potential conflict of interest.

## Supplementary Material

The Supplementary Material for this article can be found online at http://www.frontiersin.org/Journal/10.3389/fimmu.2014.00269/abstract

## References

[1] Kolenko V Wang Q Riedy MC O’Shea J Ritz J Cathcart MK Tumor-induced suppression of T lymphocyte proliferation coincides with inhibition of Jak3 expression and IL-2 receptor signaling: role of soluble products from human renal cell carcinomas. J Immunol (1997) 159(6):3057–67 9300731

[2] Mulder WM Bloemena E Stukart MJ Kummer JA Wagstaff J Scheper RJ T cell receptor-zeta and granzyme B expression in mononuclear cell infiltrates in normal colon mucosa and colon carcinoma. Gut (1997) 40(1): 113–9 9155587 10.1136/gut.40.1.113 PMC1027019

[3] Camp BJ Dyhrman ST Memoli VA Mott LA Barth RJ Jr In situ cytokine production by breast cancer tumor-infiltrating lymphocytes. Ann Surg Oncol (1996) 3(2):176–84 10.1007/BF02305798 8646519

[4] Gimmi CD Morrison BW Mainprice BA Gribben JG Boussiotis VA Freeman GJ Breast cancer-associated antigen, DF3/MUC1, induces apoptosis of activated human T cells. Nat Med (1996) 2(12):1367–70 10.1038/nm1296-1367 8946837

[5] Bennett MW O’Connell J O’Sullivan GC Brady C Roche D Collins JK The Fas counterattack in vivo: apoptotic depletion of tumor-infiltrating lymphocytes associated with Fas ligand expression by human esophageal carcinoma. J Immunol (1998) 160(11):5669–75 9605174

[6] Jewett A Head C Cacalano NA Emerging mechanisms of immunosuppression in oral cancers. J Dent Res (2006) 85(12):1061–73 10.1177/154405910608501201 17122156

[7] Tsuboi I Tanaka H Nakao M Shichijo S Itoh K Nonsteroidal anti-inflammatory drugs differentially regulate cytokine production in human lymphocytes: up-regulation of TNF, IFN-gamma and IL-2, in contrast to down-regulation of IL-6 production. Cytokine (1995) 7(4):372–9 10.1006/cyto.1995.0047 8589268

[8] Miescher S Stoeck M Qiao L Barras C Barrelet L von Fliedner V Preferential clonogenic deficit of CD8-positive T-lymphocytes infiltrating human solid tumors. Cancer Res (1988) 48(24 Pt 1):6992–8 3263897

[9] Qin J Han B Pang J The relationship between TIL from human primary hepatic carcinoma and prognosis. Zhonghua Yi Xue Za Zhi (1997) 77(3):167–70 9596951

[10] Han X Papadopoulos AJ Ruparelia V Devaja O Raju KS Tumor lymphocytes in patients with advanced ovarian cancer: changes during in vitro culture and implications for immunotherapy. Gynecol Oncol (1997) 65(3):391–8 10.1006/gyno.1997.4668 9190963

[11] Baskic D Vujanovic L Arsenijevic N Whiteside TL Myers EN Vujanovic NL Suppression of natural killer-cell and dendritic-cell apoptotic tumoricidal activity in patients with head and neck cancer. Head Neck (2013) 35(3):388–98 10.1002/hed.22968 22488918 PMC3721337

[12] Szczepanski MJ Szajnik M Welsh A Whiteside TL Boyiadzis M Blast-derived microvesicles in sera from patients with acute myeloid leukemia suppress natural killer cell function via membrane-associated transforming growth factor-beta1. Haematologica (2011) 96(9):1302–9 10.3324/haematol.2010.039743 21606166 PMC3166100

[13] Bauernhofer T Kuss I Henderson B Baum AS Whiteside TL Preferential apoptosis of CD56dim natural killer cell subset in patients with cancer. Eur J Immunol (2003) 33(1):119–24 10.1002/immu.200390014 12594840

[14] Katou F Ohtani H Watanabe Y Nakayama T Yoshie O Hashimoto K Differing phenotypes between intraepithelial and stromal lymphocytes in early-stagetongue cancer. Cancer Res (2007) 67(23):11195–201 10.1158/0008-5472.CAN-07-2637 18056444

[15] Aggarwal S Pittenger MF Human mesenchymal stem cells modulate allogeneic immune cell responses. Blood (2005) 105(4):1815–22 10.1182/blood-2004-04-1559 15494428

[16] Selmani Z Naji A Zidi I Favier B Gaiffe E Obert L Human leukocyte antigen-G5 secretion by human mesenchymal stem cells is required to suppress T lymphocyte and natural killer function and to induce CD4+CD25highFOXP3+ regulatory T cells. Stem Cells (2008) 26(1):212–22 10.1634/stemcells.2007-0554 17932417

[17] Spaggiari GM Capobianco A Abdelrazik H Becchetti F Mingari MC Moretta L Mesenchymal stem cells inhibit natural killer-cell proliferation, cytotoxicity, and cytokine production: role of indoleamine 2,3-dioxygenase and prostaglandin E2. Blood (2008) 111(3):1327–33 10.1182/blood-2007-02-074997 17951526

[18] Jewett A Bonavida B Target-induced anergy of natural killer cytotoxic function is restricted to the NK-target conjugate subset. Cell Immunol (1995) 160(1):91–7 10.1016/0008-8749(95)80013-9 7842490

[19] Jewett A Bonavida B Target-induced inactivation and cell death by apoptosis in a subset of human NK cells. J Immunol (1996) 156(3):907–15 8558016

[20] Lai P Rabinowich H Crowley-Nowick PA Bell MC Mantovani G Whiteside TL Alterations in expression and function of signal-transducing proteins in tumor-associated T and natural killer cells in patients with ovarian carcinoma. Clin Cancer Res (1996) 2(1):161–73 9816103

[21] Kuss I Saito T Johnson JT Whiteside TL Clinical significance of decreased zeta chain expression in peripheral blood lymphocytes of patients with head and neck cancer. Clin Cancer Res (1999) 5(2):329–34 10037182

[22] Patankar MS Jing Y Morrison JC Belisle JA Lattanzio FA Deng Y Potent suppression of natural killer cell response mediated by the ovarian tumor marker CA125. Gynecol Oncol (2005) 99(3):704–13 10.1016/j.ygyno.2005.07.030 16126266

[23] Jewett A Gan XH Lebow LT Bonavida B Differential secretion of TNF-alpha and IFN-gamma by human peripheral blood-derived NK subsets and association with functional maturation. J Clin Immunol (1996) 16(1):46–54 10.1007/BF01540972 8926285

[24] Jewett A Cavalcanti M Bonavida B Pivotal role of endogenous TNF-alpha in the induction of functional inactivation and apoptosis in NK cells. J Immunol (1997) 159(10):4815–22 9366406

[25] Jewett A Bonavida B MHC-class I antigens regulate both the function and the survival of human peripheral blood NK cells: role of endogenously secreted TNF-alpha. Clin Immunol (2000) 96(1):19–28 10.1006/clim.2000.4871 10873424

[26] Jewett A Cacalano NA Head C Teruel A Coengagement of CD16 and CD94 receptors mediates secretion of chemokines and induces apoptotic death of naive natural killer cells. Clin Cancer Res (2006) 12(7 Pt 1):1994–2003 10.1158/1078-0432.CCR-05-2306 16609008

[27] Jewett A Teruel A Romero M Head C Cacalano N Rapid and potent induction of cell death and loss of NK cell cytotoxicity against oral tumors by F(ab’)2 fragment of anti-CD16 antibody. Cancer Immunol Immunother (2008) 57(7):1053–66 10.1007/s00262-007-0437-6 18188563 PMC11030859

[28] Teruel A Romero M Cacalano NA Head C Jewett A Potential contribution of naive immune effectors to oral tumor resistance: role in synergistic induction of VEGF, IL-6, and IL-8 secretion. Cancer Immunol Immunother (2007) 57(3):359–66 17703300 10.1007/s00262-007-0375-3 PMC11030830

[29] Tseng HC Arasteh A Paranjpe A Teruel A Yang W Behel A Increased lysis of stem cells but not their differentiated cells by natural killer cells; de-differentiation or reprogramming activates NK cells. PLoS One (2010) 5(7):e11590 10.1371/journal.pone.0011590 20661281 PMC2905395

[30] Jewett A Cacalano NA Teruel A Romero M Rashedi M Wang M Inhibition of nuclear factor kappa B (NFkappaB) activity in oral tumor cells prevents depletion of NK cells and increases their functional activation. Cancer Immunol Immunother (2006) 55(9):1052–63 10.1007/s00262-005-0093-7 16328384 PMC11030165

[31] Jewett A Wang MY Teruel A Poupak Z Bostanian Z Park NH Cytokine dependent inverse regulation of CD54 (ICAM1) and major histocompatibility complex class I antigens by nuclear factor kappaB in HEp2 tumor cell line: effect on the function of natural killer cells. Hum Immunol (2003) 64(5):505–20 10.1016/S0198-8859(03)00039-9 12691701

[32] Pasparakis M Courtois G Hafner M Schmidt-Supprian M Nenci A Toksoy A TNF-mediated inflammatory skin disease in mice with epidermis-specific deletion of IKK2. Nature (2002) 417(6891):861–6 10.1038/nature00820 12075355

[33] Karin M Cao Y Greten FR Li ZW NF-kappaB in cancer: from innocent bystander to major culprit. Nat Rev Cancer (2002) 2(4):301–10 10.1038/nrc780 12001991

[34] Jewett A Tseng HC Tumor microenvironment may shape the function and phenotype of NK cells through the induction of split anergy and generation of regulatory NK cells. In: Shurin M Umansky V Malyguine A, editors. The Tumor Immunoenvironment. New York: Springer (2013). p. 361–84

[35] Jewett A Arasteh A Tseng HC Behel A Arasteh H Yang W Strategies to rescue mesenchymal stem cells (MSCs) and dental pulp stem cells (DPSCs) from NK cell mediated cytotoxicity. PLoS One (2010) 5(3):e9874 10.1371/journal.pone.0009874 20360990 PMC2847602

[36] Jewett A Man YG Tseng HC Dual functions of natural killer cells in selection and differentiation of stem cells; role in regulation of inflammation and regeneration of tissues. J Cancer (2013) 4(1):12–24 10.7150/jca.5519 23386901 PMC3564243

[37] Jewett A Tseng HC Potential rescue, survival and differentiation of cancer stem cells and primary non-transformed stem cells by monocyte-induced split anergy in natural killer cells. Cancer Immunol Immunother (2012) 61(2):265–74 10.1007/s00262-011-1163-7 22116348 PMC11029795

[38] Jewett A Tseng HC Arasteh A Saadat S Christensen RE Cacalano NA Natural killer cells preferentially target cancer stem cells; role of monocytes in protection against NK cell mediated lysis of cancer stem cells. Curr Drug Deliv (2012) 9(1):5–16 10.2174/156720112798375989 22023212

[39] Jewett A Bonavida B Interferon-alpha activates cytotoxic function but inhibits interleukin-2-mediated proliferation and tumor necrosis factor-alpha secretion by immature human natural killer cells. J Clin Immunol (1995) 15(1):35–44 10.1007/BF01489488 7759599

[40] Dean M Fojo T Bates S Tumour stem cells and drug resistance. Nat Rev Cancer (2005) 5(4):275–84 10.1038/nrc1590 15803154

[41] Levin TG Powell AE Davies PS Silk AD Dismuke AD Anderson EC Characterization of the intestinal cancer stem cell marker CD166 in the human and mouse gastrointestinal tract. Gastroenterology (2010) 139(6):2072.e–82.e 10.1053/j.gastro.2010.08.053 20826154 PMC2997177

[42] Pang R Law WL Chu AC Poon JT Lam CS Chow AK A subpopulation of CD26+ cancer stem cells with metastatic capacity in human colorectal cancer. Cell Stem Cell (2010) 6(6):603–15 10.1016/j.stem.2010.04.001 20569697

[43] Visvader JE Lindeman GJ Cancer stem cells in solid tumours: accumulating evidence and unresolved questions. Nat Rev Cancer (2008) 8(10):755–68 10.1038/nrc2499 18784658

[44] Leipzig ND Xu C Zahir T Shoichet MS Functional immobilization of interferon-gamma induces neuronal differentiation of neural stem cells. J Biomed Mater Res A (2010) 93(2):625–33 10.1002/jbm.a.32573 19591237

[45] el-Shami KM Tzehoval E Vadai E Feldman M Eisenbach L Induction of antitumor immunity with modified autologous cells expressing membrane-bound murine cytokines. J Interferon Cytokine Res (1999) 19(12):1391–401 10.1089/107999099312858 10638708

[46] Fremond C Allie N Dambuza I Grivennikov SI Yeremeev V Quesniaux VF Membrane TNF confers protection to acute mycobacterial infection. Respir Res (2005) 6:136 10.1186/1465-9921-6-136 16285886 PMC1325056

[47] Grönberg A Ferm MT Ng J Reynolds CW Ortaldo JR IFN-gamma treatment of K562 cells inhibits natural killer cell triggering and decreases the susceptibility to lysis by cytoplasmic granules from large granular lymphocytes. J Immunol (1988) 140(12):4397–402 3131433

[48] de Fries RU Golub SH Characteristics and mechanism of IFN-gamma-induced protection of human tumor cells from lysis by lymphokine-activated killer cells. J Immunol (1988) 140(10):3686–93 3129500

[49] Wang R Jaw JJ Stutzman NC Zou Z Sun PD Natural killer cell-produced IFN-gamma and TNF-alpha induce target cell cytolysis through up-regulation of ICAM-1. J Leukoc Biol (2012) 91(2):299–309 10.1189/jlb.0611308 22045868 PMC3290424

[50] Jewett A Man YG Tseng HC Dual functions of natural killer cells in selection and differentiation of stem cells; role in regulation of inflammation and regeneration of tissues. J Cancer (2013) 4(1):12–24 10.7150/jca.5519 23386901 PMC3564243

