# Supplementary material for: Induction of Split Anergy Conditions Natural Killer Cells to Promote Differentiation of Stem Cells through Cell–Cell Contact and Secreted Factors
Source: Front Immunol. 2014 Jun 19;5:269. doi: 10.3389/fimmu.2014.00269 (PMC4062968; doi:10.3389/fimmu.2014.00269)
Supplement: Supplementary file 1 [file Presentation1.PDF]

Supplemental data;

Induction of resistance in MP2 and SCAPs to NK cell mediated cytotoxicity by anergized NK cells is mediated by the combination of IFN-γ and TNF-α and not each cytokine alone

Similar results to those obtained with OSCSCs were also observed when undifferentiated or stem-like Mia Paca (MP2) pancreatic tumors or Stem Cell of Apical Papillae (SCAP) were used to treat with the supernatants of NK cells treated with IL-2+anti-CD16mAb in the presence and absence of anti-TNF-α and/or anti-IFN-γ (Fig. 1). Reversal or inhibition of resistance of MP2 (Fig. 1A) and SCAP (Fig. 1B) to NK cell mediated cytotoxicity correlated with the ability of the combination of anti-TNF-α and anti-IFN-γ to block up-regulation of CD54, MHC-class I and B7H1 and down-regulation of CD44 (Fig. 1C and 1D). No or slight modulation of CD54 could be observed on MP2 cells with all the treatments. The effect of anti-IFN-γ mAb in the absence of anti-TNF-α antibody was more dominant for surface receptor expression than cytotoxicity or cell growth since its addition abrogated the increase in surface receptor expression substantially. Similarly, the rate of MP2 (Fig. 1E) and SCAP (Fig. 1G) cell growth was decreased when supernatants obtained from IL-2+anti-CD16 treated NK cells were added, and this decrease was completely inhibited in the presence of the combination of anti-IFN-γ and anti-TNF-α antibodies and not each antibody alone. A slight increase in cell death of MP2 (Fig. 1F) or SCAP (Fig. 1H) could be observed in both attached and detached cells when these cells were treated with the supernatants from IL-2+anti-CD16mAb treated NK cells, however, in contrast to OSCSCs, MP2 and SCAPs had substantially less detached cells, and required higher

amounts of NK supernatants for their differentiation as indicated in the materials and methods section.

Combination of rTNF-α and rIFN-γ induce differentiation and resistance of DPSCs and SCAPs to NK cell mediated cytotoxicity

Similar results to those obtained with OSCSCs were also obtained with SCAPs and DPSCs, although rIFN-γ reduced cytotoxicity by IL-2 activated NK cells, the addition of a combination of rIFN-γ and rTNF-α further reduced IL-2 mediated cytotoxicity (Fig. 2A). Interestingly, the level of CD44 was augmented by the treatment of rTNF-α and rIFN-γ and their combination, and the addition of anti-TNF-α and anti-IFN-γ antibodies blocked the increase substantially on DPSCs (Fig. 2B).

Figure Legends.

Fig. 1. Induction of resistance of MP2 and SCAPs to NK cell mediated cytotoxicity and inhibition of their growth by IL-2+anti-CD16mAb treated NK cells is mediated by the combination of IFN-γ and TNF-α and not each cytokine alone

Highly purified NK cells were left untreated or treated with the combination of IL-2 (1000 units/ml) and anti-CD16 mAb (3 μg/ml) for 24 hours, after which the same amounts of supernatants from different NK cell treatments were removed and added to MP2 **(A)** and SCAP **(B)** in the presence and absence of anti-TNF-α (1:100) and/or anti-IFN-γ (1:100) for a period of 7 days for MP2 and SCAP. The cytotoxicity against untreated and NK supernatant treated MP2 and SCAP in the presence of antibodies to freshly isolated untreated NK cells or IL-2 treated (1000units/ml) NK cells were assessed using a standard 4 hour ^{51}Cr release assay. Percent cytotoxicity was obtained at different effector to target ratio, and the lytic units 30/10^6 cells were determined using inverse number of NK cells required to lyse 30% of the tumor cells X100. The surface expression of CD54, CD44, B7H1 and MHC Class 1 on untreated and NK supernatant treated MP2 **(C)** and SCAP **(D)** were assessed after PE conjugated antibody staining using flow cytometric analysis. Isotype control antibodies were used as controls. The numbers on the right hand corner are the percentages and the mean channel fluorescence intensities for each histogram. At the end of the incubation of MP2 cells **(E and F)** or SCAP cells **(G and H)** with NK cell supernatants, cells which were remained attached to the plate and those which detached during the incubation period were collected separately, and the number of cells **(E and G)** and their viability **(F and H)** were assessed using microscopy, and propidium iodide staining followed by flow cytometric analysis respectively.

Fig. 2. Combination of rTNF-α and rIFN-γ induce differentiation and resistance of SCAPs and DPSCs

SCAPs **(A)** were left untreated or treated with recombinant human TNF-α (20 ng/ml), recombinant human IFN-γ (50 units/ml) or the combination of human TNF-α (20ng/ml) and recombinant human IFN-γ (50 units/ml) in the presence or absence of antibodies against TNF-α (1:100) and/or IFN-γ (1:100) for 24 hours. Afterwards, the cells were detached from the tissue culture plates and labeled with ^{51}Cr and used in a standard 4 hour chromium release assay against untreated and IL-2 (1000units/ml) treated NK cells. Pretreatment of NK cells with IL-2 were carried out for 18-24 hours. Percent cytotoxicity was determined at different effector to target ratio, and the lytic units $30/10^6$ cells were determined using inverse number of NK cells required to lyse 30% of the tumor cells X100. Surface expressions of CD54, CD44, B7H1 and MHC-1 on DPSCs **(B)** treated as described in Fig. 2A were determined using staining with PE conjugated antibodies followed by flow cytometric analysis. Isotype control antibodies were used as controls. The numbers on the right hand corner are the percentage and the mean channel fluorescence intensities in each histogram. One of minimum three representative experiments is shown in each of Fig. 2A-2B.

Fig. 1A

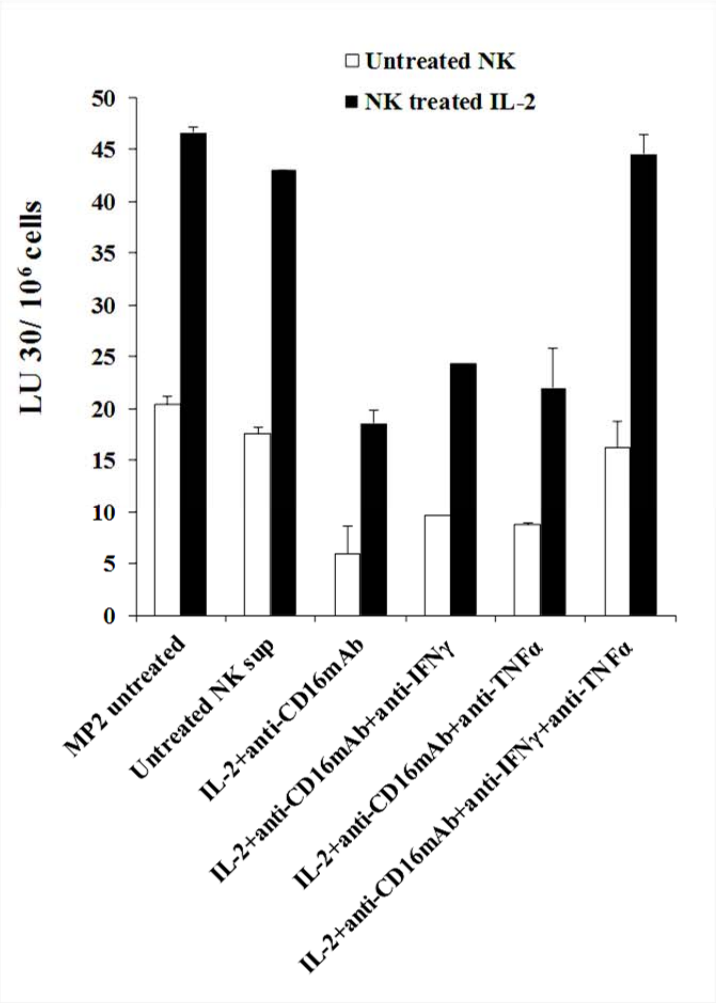

Untreated NK
NK treated IL-2
LU 30/ 10^6 cells
0
5
10
15
20
25
30
35
40
45
50
MP2 untreated
Untreated NK sup
IL-2+anti-CD16mAb
IL-2+anti-CD16mAb+anti-IFNγ
IL-2+anti-CD16mAb+anti-TNFα
IL-2+anti-CD16mAb+anti-IFNγ+anti-TNFα

Fig. 1B

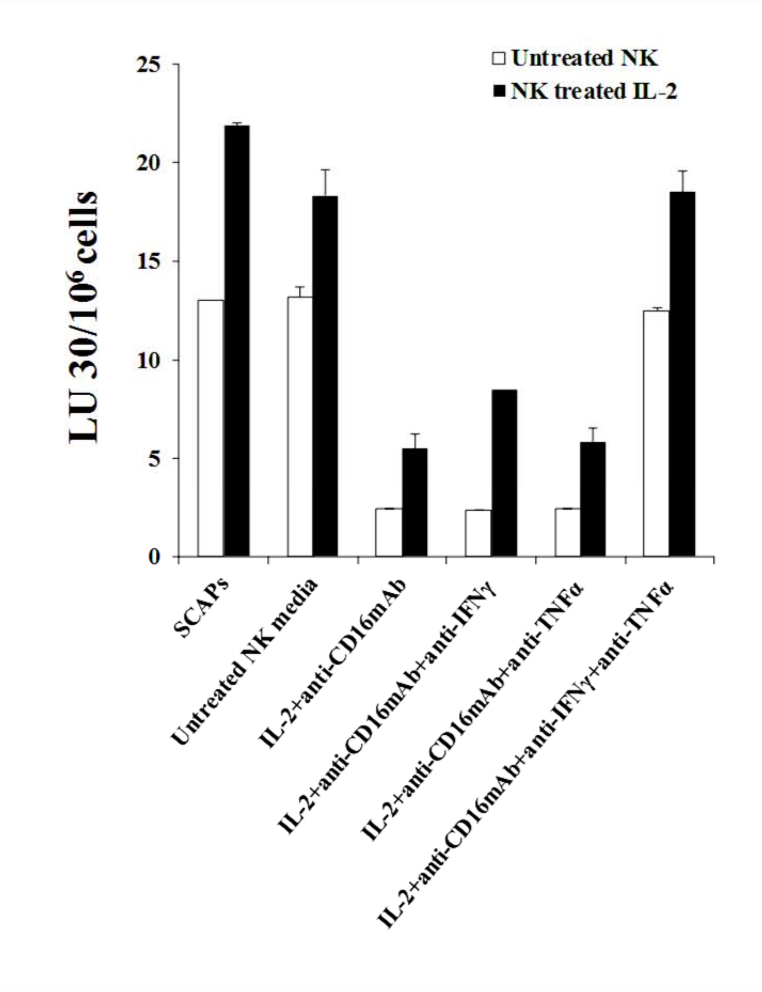

Untreated NK
NK treated IL-2
LU 30/10^6 cells
0
5
10
15
20
25
SCAPs
Untreated NK media
IL-2+anti-CD16mAb
IL-2+anti-CD16mAb+anti-IFNγ
IL-2+anti-CD16mAb+anti-TNFα
IL-2+anti-CD16mAb+anti-IFNγ+anti-TNFα

Fig. 1C

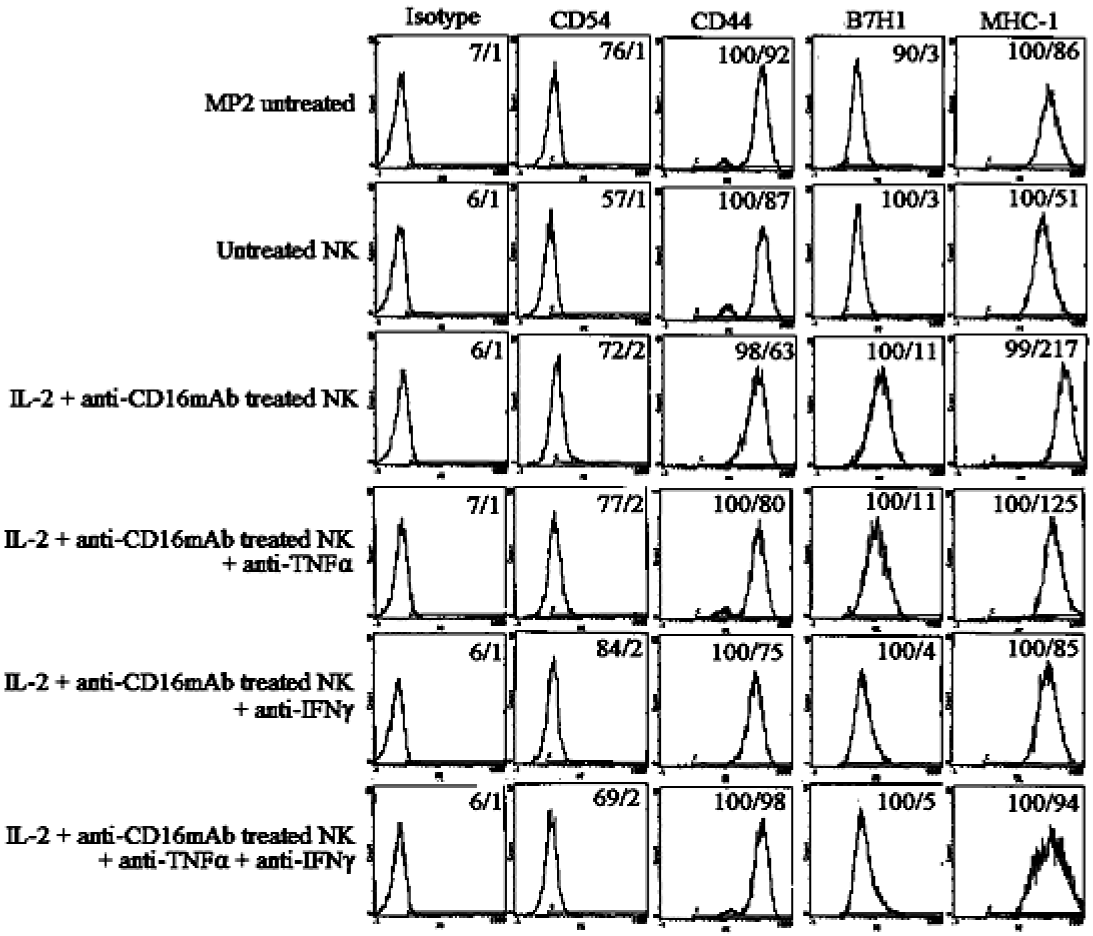

Isotype
CD54
CD44
B7H1
MHC-1
MP2 untreated
7/1
76/1
100/92
90/3
100/86
Untreated NK
6/1
57/1
100/87
100/3
100/51
IL-2 + anti-CD16mAb treated NK
6/1
72/2
98/63
100/11
99/217
IL-2 + anti-CD16mAb treated NK + anti-TNFα
7/1
77/2
100/80
100/11
100/125
IL-2 + anti-CD16mAb treated NK + anti-IFNγ
6/1
84/2
100/75
100/4
100/85
IL-2 + anti-CD16mAb treated NK + anti-TNFα + anti-IFNγ
6/1
69/2
100/98
100/5
100/94

Fig. 1D

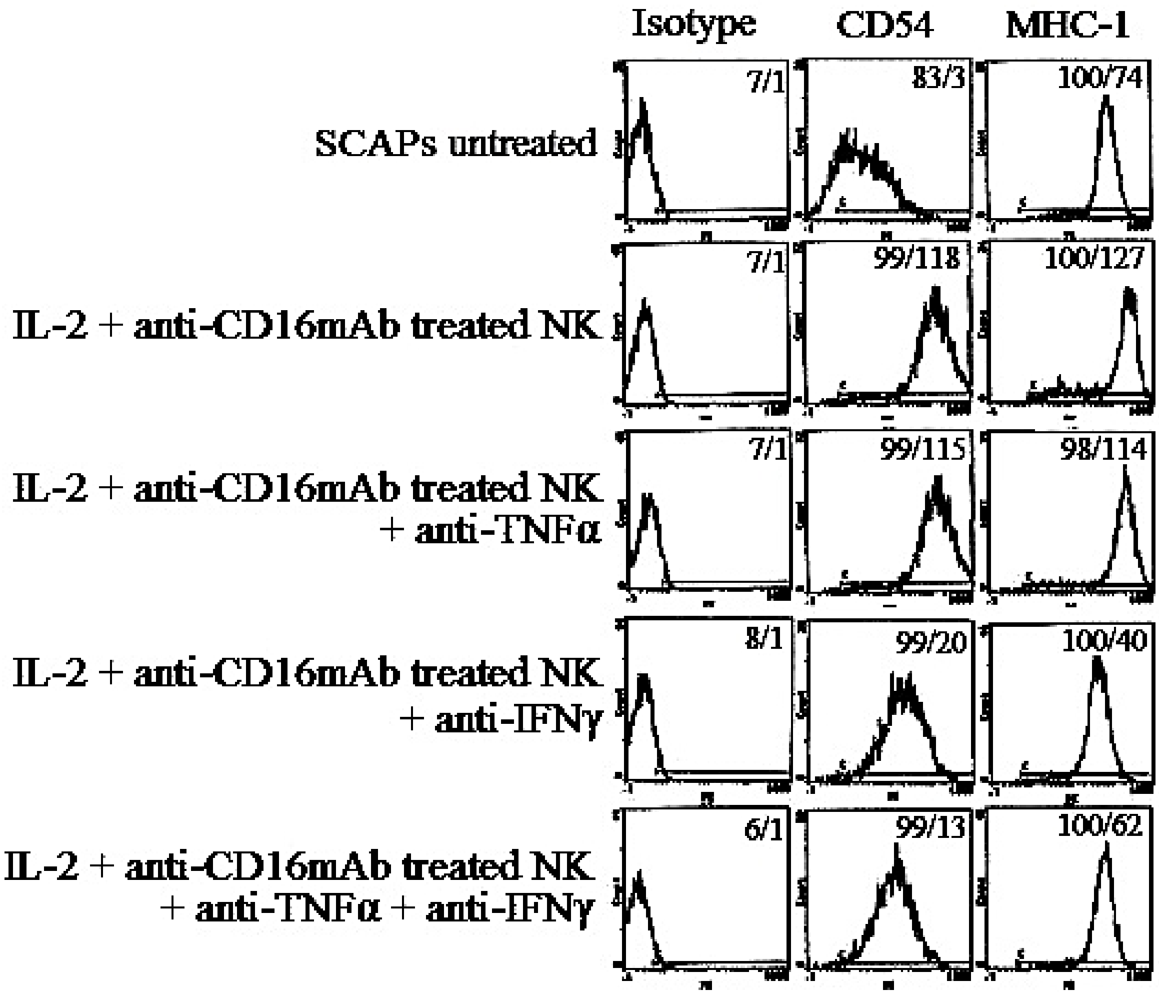
Isotype
CD54
MHC-1
SCAPs untreated
7/1
83/3
100/74
IL-2 + anti-CD16mAb treated NK
7/1
99/118
100/127
IL-2 + anti-CD16mAb treated NK + anti-TNFα
7/1
99/115
98/114
IL-2 + anti-CD16mAb treated NK + anti-IFNγ
8/1
99/20
100/40
IL-2 + anti-CD16mAb treated NK + anti-TNFα + anti-IFNγ
6/1
99/13
100/62

Fig. 1E

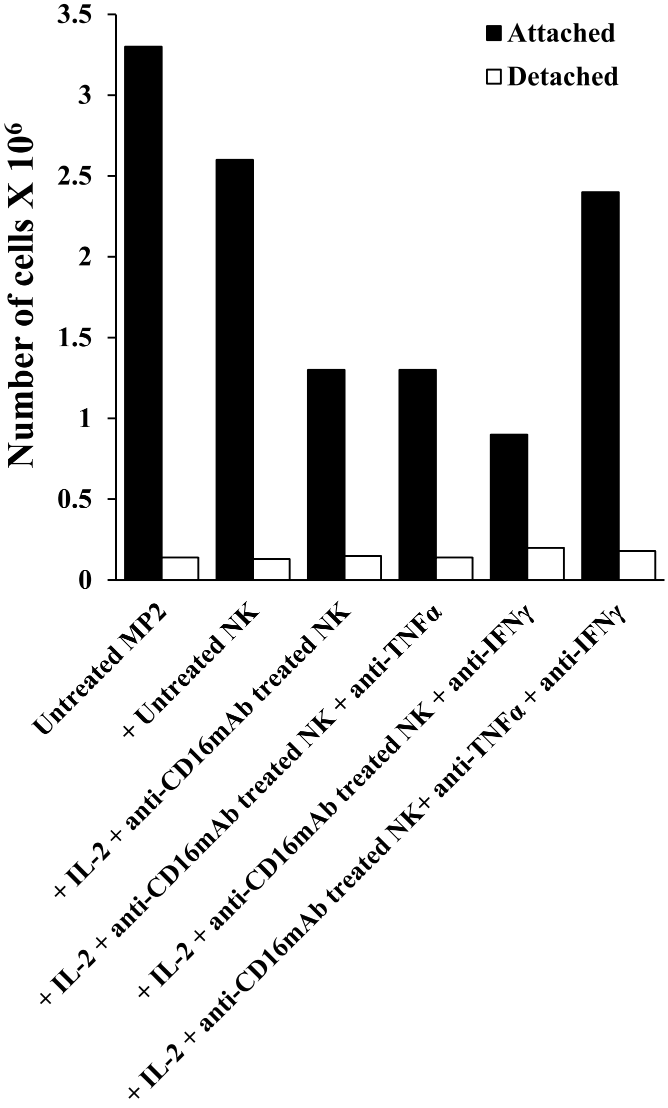
3.5
3
2.5
2
1.5
1
0.5
0
Number of cells X 10^6
Attached
Detached
Untreated MP2
+ Untreated NK
+ IL-2 + anti-CD16mAb treated NK
+ IL-2 + anti-CD16mAb treated NK + anti-TNFα
+ IL-2 + anti-CD16mAb treated NK + anti-IFNγ
+ IL-2 + anti-CD16mAb treated NK+ anti-TNFα + anti-IFNγ

Fig. 1F

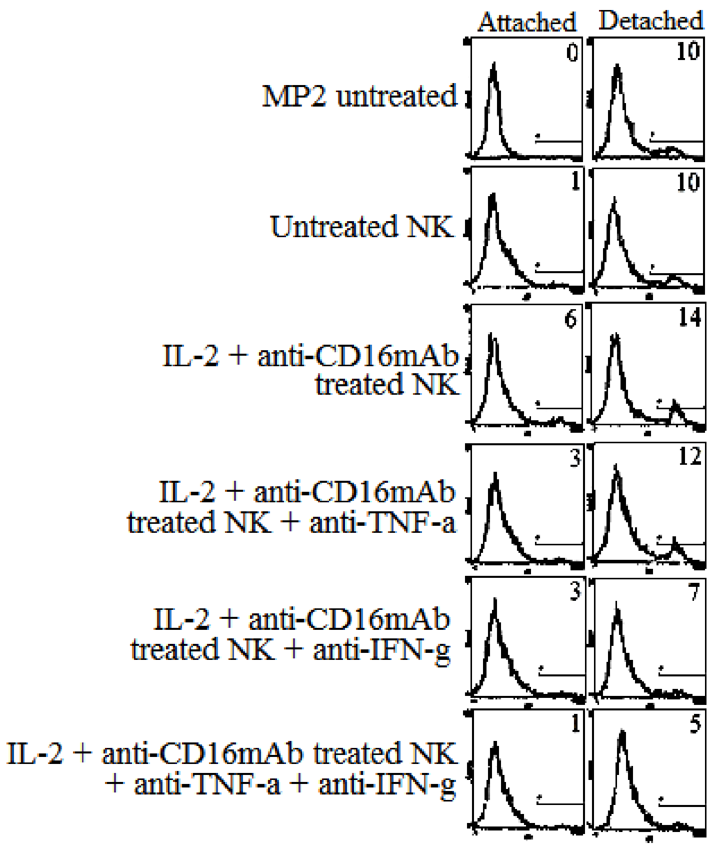
Attached
Detached
MP2 untreated
0
10
Untreated NK
1
10
IL-2 + anti-CD16mAb treated NK
6
14
IL-2 + anti-CD16mAb treated NK + anti-TNF-a
3
12
IL-2 + anti-CD16mAb treated NK + anti-IFN-g
3
7
IL-2 + anti-CD16mAb treated NK + anti-TNF-a + anti-IFN-g
1
5

Fig. 1G

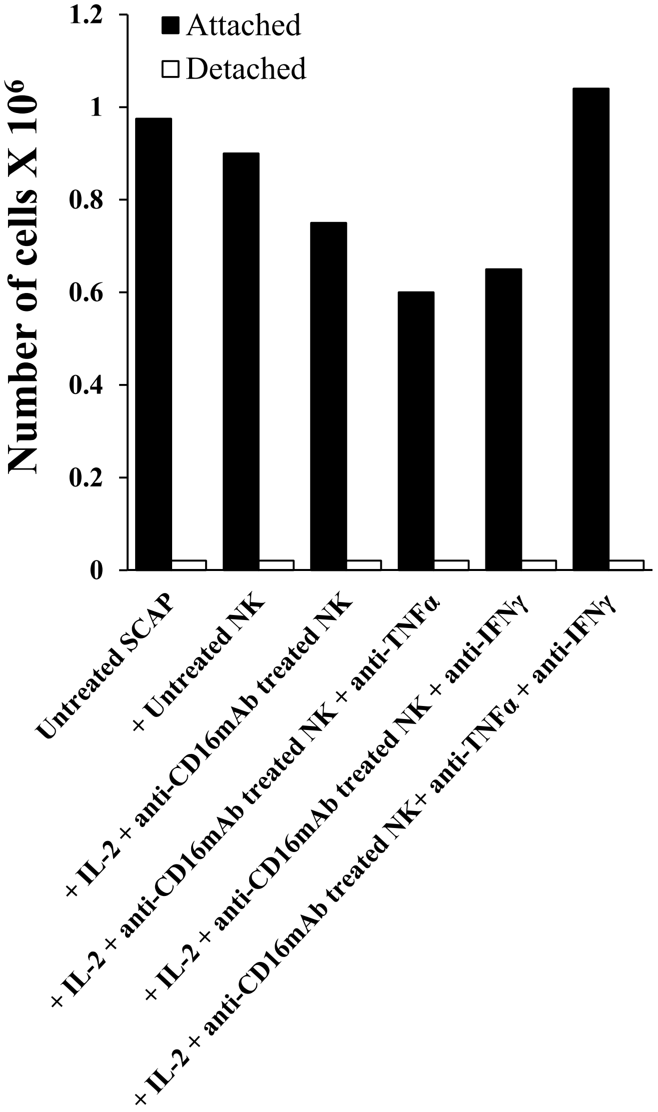

Number of cells X 10⁶
1.2
1
0.8
0.6
0.4
0.2
0
Attached
Detached
Untreated SCAP
+ Untreated NK
+ IL-2 + anti-CD16mAb treated NK
+ IL-2 + anti-CD16mAb treated NK + anti-TNFα
+ IL-2 + anti-CD16mAb treated NK + anti-IFNγ
+ IL-2 + anti-CD16mAb treated NK+ anti-TNFα + anti-IFNγ

Fig. 1H

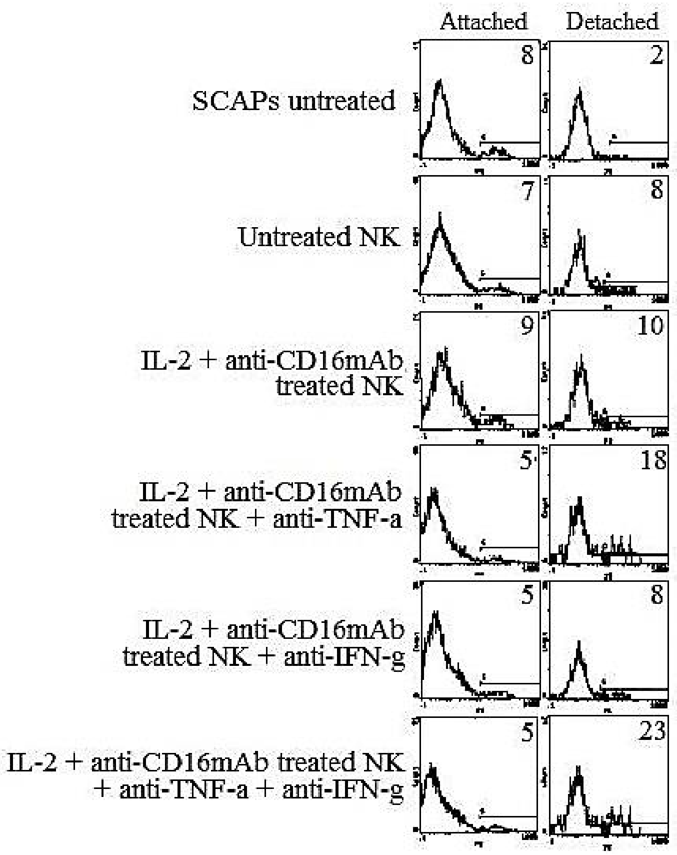

Attached
Detached
SCAPs untreated
8
2
Untreated NK
7
8
IL-2 + anti-CD16mAb treated NK
9
10
IL-2 + anti-CD16mAb treated NK + anti-TNF-a
5
18
IL-2 + anti-CD16mAb treated NK + anti-IFN-g
5
8
IL-2 + anti-CD16mAb treated NK + anti-TNF-a + anti-IFN-g
5
23

Fig. 2A

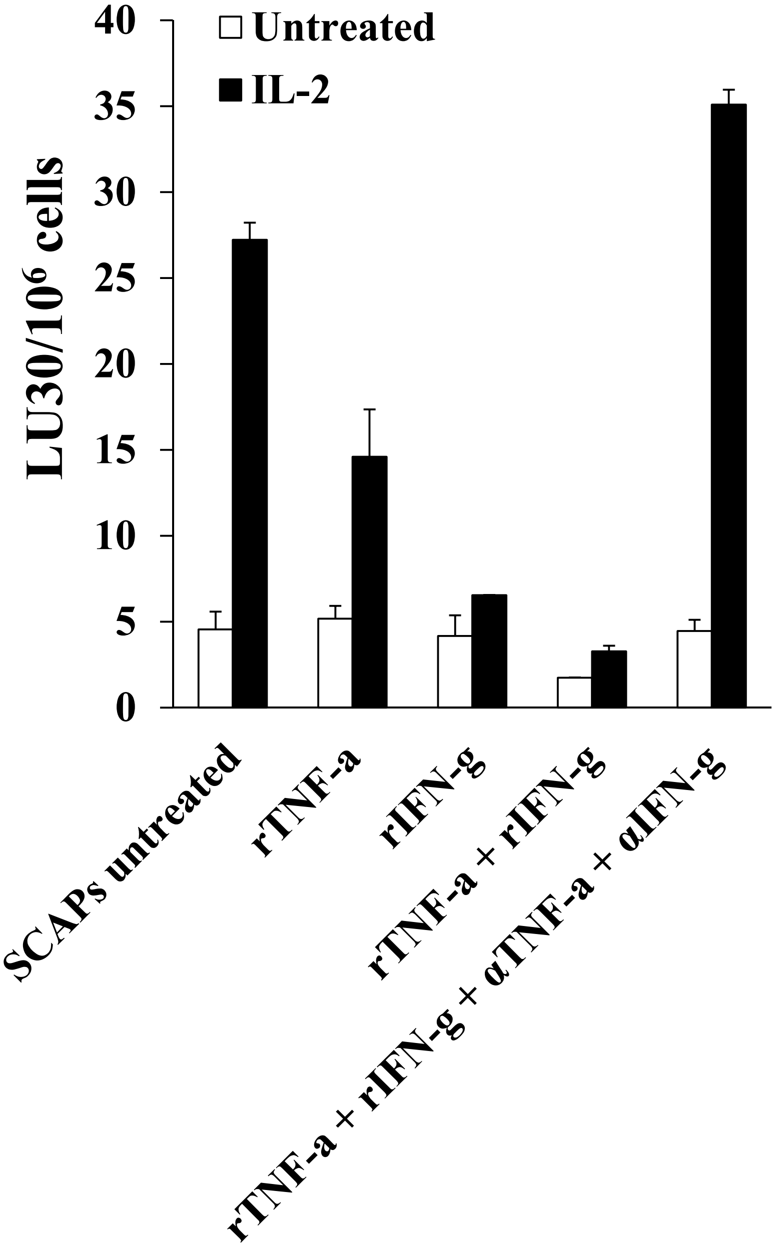

40
35
30
25
20
15
10
5
0
LU30/10^6 cells
Untreated
IL-2
SCAPs untreated
rTNF-a
rIFN-g
rTNF-a + rIFN-g
rTNF-a + rIFN-g + αTNF-a + αIFN-g

Fig. 2B

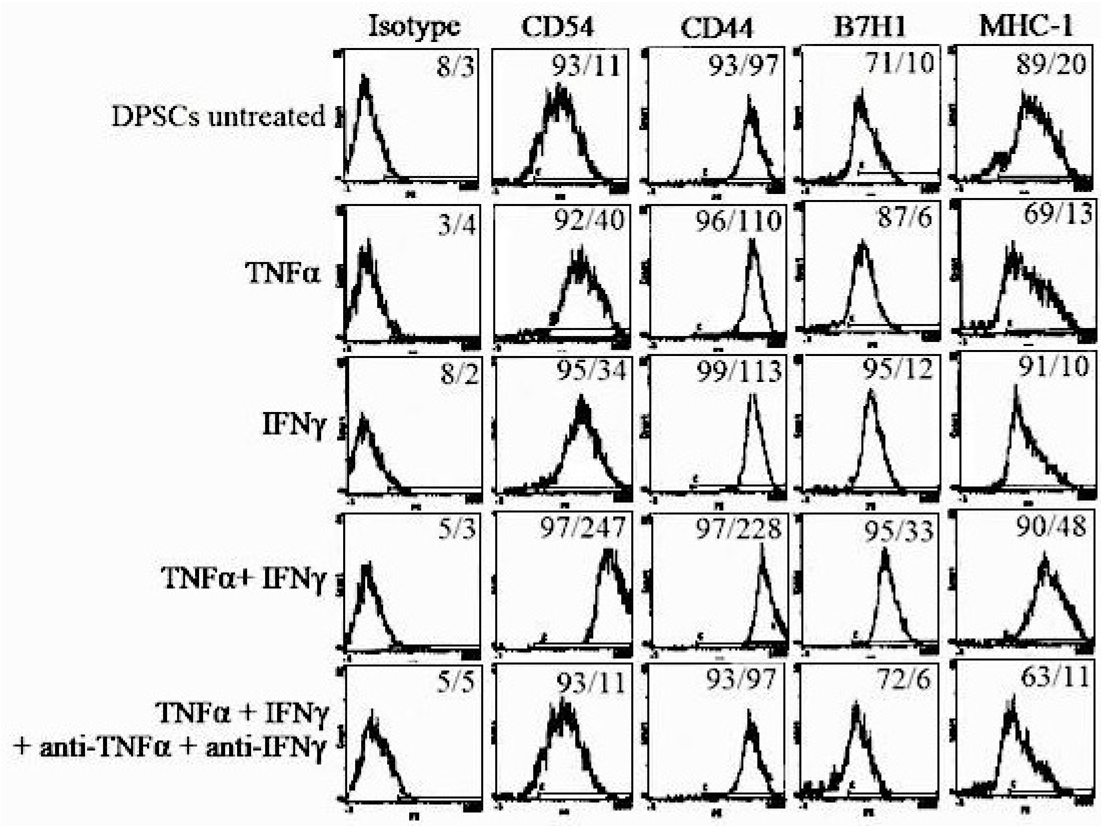
Isotype
CD54
CD44
B7H1
MHC-1
DPSCs untreated
8/3
93/11
93/97
71/10
89/20
TNFα
3/4
92/40
96/110
87/6
69/13
IFNγ
8/2
95/34
99/113
95/12
91/10
TNFα+ IFNγ
5/3
97/247
97/228
95/33
90/48
TNFα + IFNγ
+ anti-TNFα + anti-IFNγ
5/5
93/11
93/97
72/6
63/11
